# Structure‐conditioned amino‐acid couplings: How contact geometry affects pairwise sequence preferences

**DOI:** 10.1002/pro.4280

**Published:** 2022-02-15

**Authors:** Jack Holland, Gevorg Grigoryan

**Affiliations:** ^1^ Department of Computer Science Dartmouth College Hanover New Hampshire USA

**Keywords:** contact potential, coupling energy, sequence–structure relationships, statistical energy, structural modeling, tertiary motifs

## Abstract

Relating a protein's sequence to its conformation is a central challenge for both structure prediction and sequence design. Statistical contact potentials, as well as their more descriptive versions that account for side‐chain orientation and other geometric descriptors, have served as simplistic but useful means of representing second‐order contributions in sequence–structure relationships. Here we ask what happens when a pairwise potential is conditioned on the fully defined geometry of interacting backbones fragments. We show that the resulting structure‐conditioned coupling energies more accurately reflect pair preferences as a function of structural contexts. These structure‐conditioned energies more reliably encode native sequence information and more highly correlate with experimentally determined coupling energies. Clustering a database of interaction motifs by structure results in ensembles of similar energies and clustering them by energy results in ensembles of similar structures. By comparing many pairs of interaction motifs and showing that structural similarity and energetic similarity go hand‐in‐hand, we provide a tangible link between modular sequence and structure elements. This link is applicable to structural modeling, and we show that scoring CASP models with structured‐conditioned energies results in substantially higher correlation with structural quality than scoring the same models with a contact potential. We conclude that structure‐conditioned coupling energies are a good way to model the impact of interaction geometry on second‐order sequence preferences.

## 1 INTRODUCTION

For many decades now, the wealth of structural information in the Protein Data Bank (PDB) has enabled protein scientists to infer relationships between the amino‐acid sequence of a protein and its native structure based on statistical patterns. A classic example of how even simple structural statistics can provide useful information is the contact potential, which encodes the relative interaction preferences for each amino‐acid pair based on simple log‐odds ratios of observed versus expected occurrences in a large set of contacts, sometimes partitioning the set of contacts into bins according to distance or other geometric parameters.^1^, ^2^, ^3^, ^4^, ^5^, ^6^, ^7^, ^8^, ^9^, ^10^, ^11^ Contact potentials in varying forms have provided insight into sequence–structure relationships since the 1970s and have been incorporated into numerous effective predictive models over the years (e.g., Rosetta,^12^, ^13^ RaptorX,^14^ PoPMuSiC,^15^, ^16^ and many others^17^, ^18^, ^19^, ^20^, ^21^). The continued efficacy of contact potentials, despite their apparent simplicity, suggests that elaborations or extensions to the core concept may also serve as useful bridges between native sequence and structure elements. Multiple extensions have already been proposed, seeking to condition amino‐acid pair preferences on more detailed structural circumstances.^8^, ^9^, ^22^ For example, there are potentials that incorporate the relative orientation between residue pairs,^23^, ^24^, ^25^, ^26^, ^27^, ^28^ condition on residue depth to capture the effects of polarity and hydrophobicity,^29^, ^30^ include additional terms from pseudo‐physical force fields,^31^ alter the definition of contact,^32^, ^33^ and optimize parameters by contrasting the statistics of native structures and decoys.^25^, ^34^

Given the complex geometry of an inter‐residue contact and its surrounding structural context (e.g., a pair of contacting residues and their flanking residues comprises 24 backbone atoms and thus 72 spatial coordinates), there is a potentially large and high dimensional interaction space throughout which amino‐acid pair preferences might vary. In order to explore how pairwise sequence preferences might depend on interaction geometry, a more general formulation of structure conditioning is required. Such a formulation should be able to quantify the pairwise sequence preferences of any type of interaction. Just as a contact potential quantifies which amino‐acid pairs prefer to interact over a set of contacts in general, a structure‐conditioned potential (SCP) could quantify which amino‐acid pairs prefer to interact over a particular set of structurally similar contacts. Here, we define such a potential as one that takes an additional argument, an input fragment centered around a pair of contacting residues, which determines the contact geometry that the resulting statistics are conditioned on. In particular, a given input fragment is used as the query motif of a structural search which returns an ensemble of structurally similar motifs whose amino‐acid pair statistics are then used to compute the statistical energies. By conditioning on an ensemble of similar interaction motifs, there is no need to determine which geometric parameters (distances, orientations, etc.) are best suited for capturing interaction preferences, instead letting the statistics of the PDB inform the preferences via an ensemble of motifs. This process is made feasible not just by the growing size of the PDB but by the recent availability of structural search tools,^35^ which enable us to specify a query motif (i.e., a structural fragment) and efficiently search for all structurally similar fragments in a database of structures.

The primary purpose of this work is to establish a framework for understanding modular sequence–structure relationships on a pairwise level. First, we show that structure‐conditioned coupling energies (SCEs, 20 × 20 per motif) converge to similar energies as those encoded in a traditional contact potential when many interaction instances are averaged. Having established this link, we then show that SCE matrices encode more information linking the structures of pair motifs to their associated amino‐acid pairs and better reflect experimentally determined inter‐residue coupling values. Looking more deeply into the link these SCE matrices provide between structure and sequence, we show that structurally similar pair motifs are more likely to be energetically similar and the reverse, that energetically similar pair motifs are also more likely to be structurally similar. This link reveals the modular and context sensitive link between sequence and structure elements and we demonstrate a general relationship between the two. Turning to structure modeling, we compare how well SCEs can evaluate the structural quality of CASP models to how well contact potentials do so, highlighting how much additional information is encoded by conditioning on structure. As simple, interpretable objects that still offer valuable information on second‐order contributions to sequence–structure relationships, we find the SCP to be a convenient tool for dissecting the sequential and energetic effects of interaction geometry.

Recent breakthrough successes in structure prediction, with end‐to‐end deep learning models now able to give accurate predictions of a sequence's native contacts^36^ and structure,^37^, ^38^ suggest that the PDB contains many generalizable patterns that relate sequence and structure. The SCP is an example of a PDB‐derived generalization, being simple to describe and understand while capturing important aspects of pairwise contributions to sequence–structure relationships in a context‐sensitive way. Better understanding these and other generalizations will be helpful for guiding novel prediction and design methods, especially in areas where improved performance is much needed, such as the prediction of protein–protein interfaces.^39^

## 2 RESULTS

### 2.1 Definitions of the contact potential and structure‐conditioned potential

A contact potential infers pseudo‐energies associated with amino‐acid pair interactions from observations of amino‐acid contacts in a structural database. In its simplest form, a contact potential measures the extent each amino‐acid pair is over‐ or under‐observed relative to the number of observations expected if there were no pair preferences^3^ (e.g., the number expected based on the product of the marginal distributions of both amino acids comprising the pair). If the database from which the amino‐acid pair statistics arise includes a large diversity of contacts, the resulting pseudo‐energies reflect how disproportionately each amino‐acid pair interacts in native structural contexts. For example, cysteine‐cysteine contacts are observed much more frequently than expected based on the low background frequency of cysteine residues in native structures and therefore cysteine‐cysteine contacts have a strongly negative (favorable) energy according to a traditionally derived contact potential. There are alternative formulations of the reference state—the framework for computing the expected number of observations^7^, ^9^, ^25^—but most assume an independence between sequence and structure elements and therefore estimate cysteine‐cysteine interactions favorably. Equation (1) describes the traditional formulation of a contact potential, where *N*_obs_(*a*) is the number of observations in the database of pairs of amino‐acid type *a*, *N*_obs_(*a*, *b*) is the number of observations of amino‐acid types *a* and *b* in contact in either order, and *N* is the total number of amino acids in the database of pairs. The term *H*(*a*, *b*) adjusts the expectation for heterotypic pairs (i.e., amino‐acid pairs for which *a* is not *b*) as the potential is directionless. The pseudocount *ε* ensures sparse statistics do not result in a division of or by zero. Taking the negative log of the ratio transforms the values into additive pseudo‐energies. See Section 4.4 for details.

(1) $$E\left(a,b\right)=E\left(b,a\right)=-\log\frac{N_{\mathrm{obs}}\left(a,b\right)+\varepsilon }{N_{\exp}\left(a,b\right)+\varepsilon }=-\log\frac{N_{\mathrm{obs}}\left(a,b\right)+\varepsilon }{N_{\mathrm{obs}}\left(a\right)\cdot N_{\mathrm{obs}}\left(b\right)\cdot H\left(a,b\right)/N+\varepsilon }.$$

The SCP computes a contact potential for a given interaction motif (i.e., a specific pair of residues together with their surrounding backbone fragments). The resulting interaction matrix encodes the pseudo‐energetic preferences for each amino‐acid pair in this given structural context. Like a contact potential, these pseudo‐energetic preferences are calculated from amino‐acid pair statistics, encoding to what extent each amino‐acid pair is over‐ or under‐observed relative to the number expected if the two positions in question did not influence each other. Unlike a contact potential, the amino‐acid statistics do not come from a generic database of contacts, but instead from a structural ensemble of fragments that share a similar geometry with the interaction motif in question (i.e., constrained by a maximum RMSD over backbone atoms to the input motif). The resulting SCEs reflect the extent to which each amino‐acid pair disproportionately interacts in the specific structural context of the interaction motif. The formulation of SCEs is therefore similar to Equation 1 but computed over a constrained set of statistics:

(2) $$\mathrm{SCE}\left(a,b\right)=-\log\frac{N_{\mathrm{obs}}\left(a,b\right)+\varepsilon }{N_{\exp}\left(a,b\right)+\varepsilon }.$$

Here, (*a*, *b*) is the amino‐acid pair, *N*_obs_(*a*, *b*) is the number of occurrences of pair (*a*, *b*) in the interaction motif's ensemble of matching fragments, *N*_exp_(*a*, *b*) is the number of occurrences of pair (*a*, *b*) that would be expected if there were no pair preferences, and *ε* is a pseudocount. See Section 4.3 for details.

**FIGURE 1:**
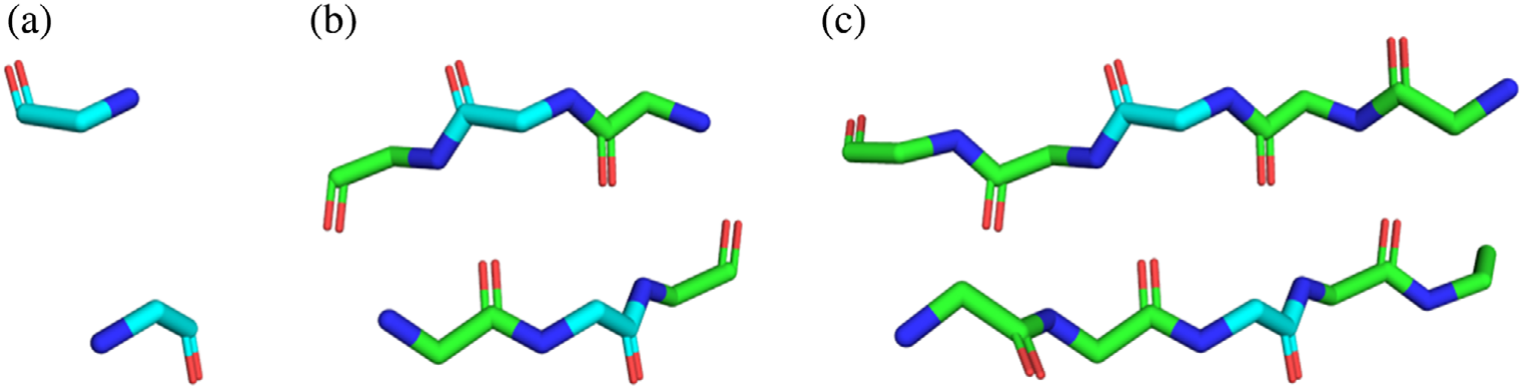
Visualization of an interaction motif. (a–c) The same pair of interacting residues is shown with increasing structural context: 1 × 1 (a), 3 × 3 (b), and 5 × 5 (c). In each case, the pair of interacting residues is colored in cyan. The pair of interacting residues is (A108, A188) from the structure with PDB ID 4G1Q and was visualized in PyMOL^40^

In order to measure the impact of incorporating additional structural context, here we consider three types of interaction motifs that incorporate an increasing number of flanking residues around a pair of interacting positions. Specifically, 1 × 1, 3 × 3, and 5 × 5 motifs comprise a pair of contacting residues with no flanking residues, one flanking residue on each side, or two flanking residues on each side, respectively (Figure 1). Larger motifs may carry important contextual information, which can offer certain advantages, but may also be associated with decreased statistics in a limited database. Comparing the results with these different motif types measures the impact of increased structural context and potentially decreased database statistics.

### 2.2 Averaging SCEs over many structural contexts converges to a traditional contact potential

**FIGURE 2:**
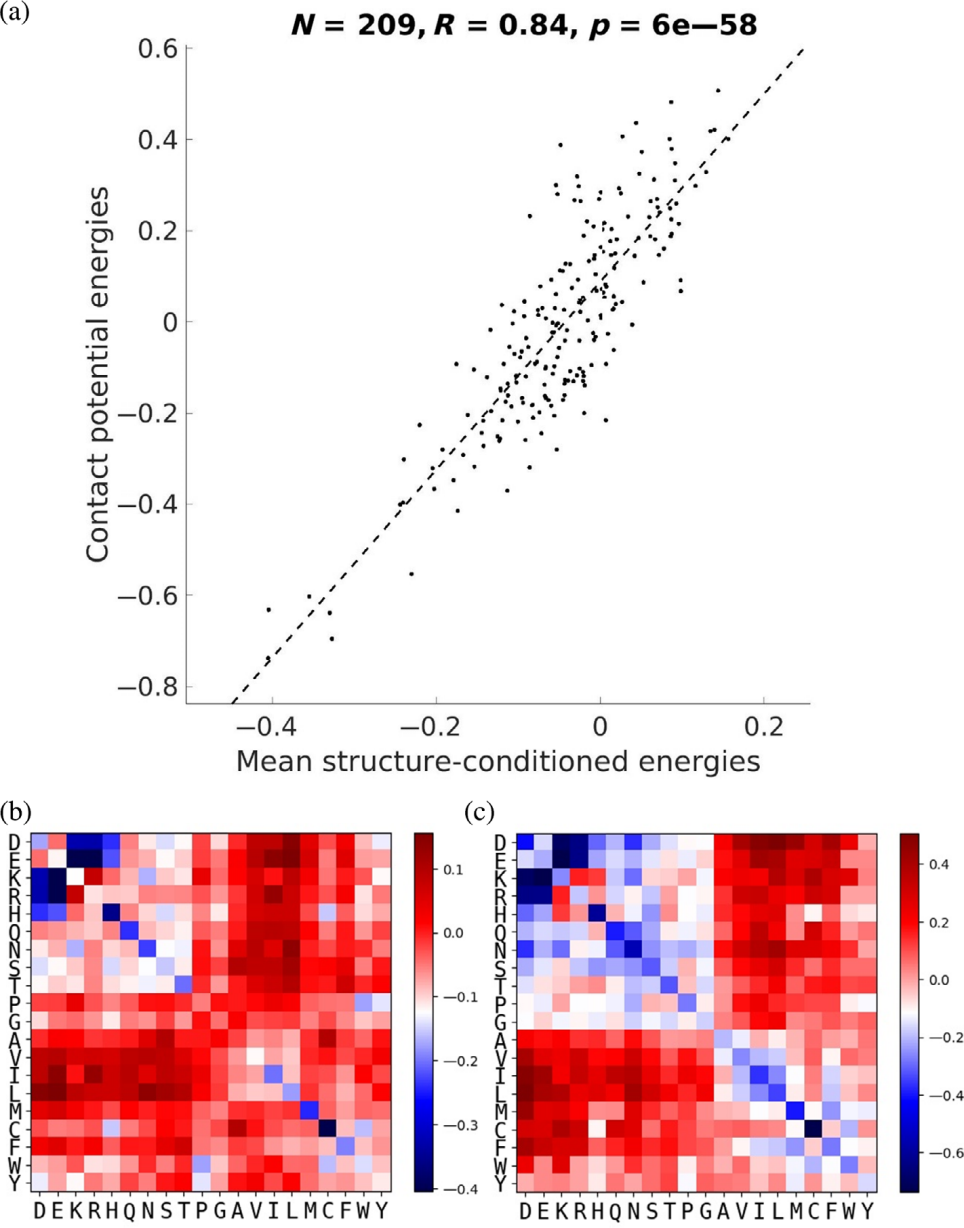
Correlation between each SCE from 3 × 3 motifs, averaged over many contexts, and the corresponding energy according to a contact potential. (a) Mean SCEs plotted against corresponding contact‐potential energies. Pair Cys‐Cys is not shown as it occupies a point far to the bottom‐left (contact potential of −1.78 and mean SCE of −1.10), though its inclusion increases the correlation to *R* = 0.88. The dotted line indicates the best linear fit of the data. (b) Heatmap of the mean SCEs. (c) Heatmap of contact‐potential energies. For (b) and (c), the energy scale was capped at the most favorable energy except for Cys‐Cys for easier visualization

If each SCE matrix encodes the amino‐acid pair energies in a particular context, then the average of each energy over many contexts should encode similar information to a generic contact potential. To test this, a database of 200,000 contacts from a nonredundant subset of the PDB (DB200K, see Section 4.2) was used to compute a contact potential and, for each of their corresponding 3 × 3 interaction motifs, a 20 × 20 matrix of SCEs. For each amino‐acid pair, the average SCE over all contacts involving the pair in the database was computed. Figure 2 plots contact potential energies (CEs) against corresponding mean SCEs, showing a linear correlation coefficient of *R* = 0.88. In contrast, SCEs at a particular pair of sites generally correlate quite poorly with contact potential energies (*R* = 0.20 on average, see Figure S1A). This suggests that while SCEs and CEs capture similar effects, and converge on average, SCEs are much more context sensitive and thus have the potential to capture many details that CEs may miss.

Interestingly, the strong correlation between mean SCEs and CEs also holds for 1 × 1 and 5 × 5 motifs but does show a decline with increased context length (i.e., *R* = 0.91 and *R* = 0.84 for 1 × 1 and 5 × 5, respectively; Figure S1B,C). This decline is expected as more averaging would be needed to integrate out the influence of more detailed structural context. Overall, these results show that this SCP can be thought of as a more elaborate and context‐sensitive counterpart of the traditional contact potential.

### 2.3 Conditioning on structure encodes more accurate sequence information

A simple test of the information gained by conditioning on structural context is to measure how reliably the SCP favors the native amino‐acid pair of an interaction motif it corresponds to, compared to how reliably the contact potential favors it. Note that one may not expect very high performance in such a test, as the native choice of amino‐acid pairs is guided not only by second‐order effects, but also (and perhaps more importantly) by first‐order effects. Nevertheless, as a vehicle for discerning the effects of structure conditioning and context, the test is still fair—that is, a more accurate second‐order potential *should* more frequently pick out the native pair.

**FIGURE 3:**
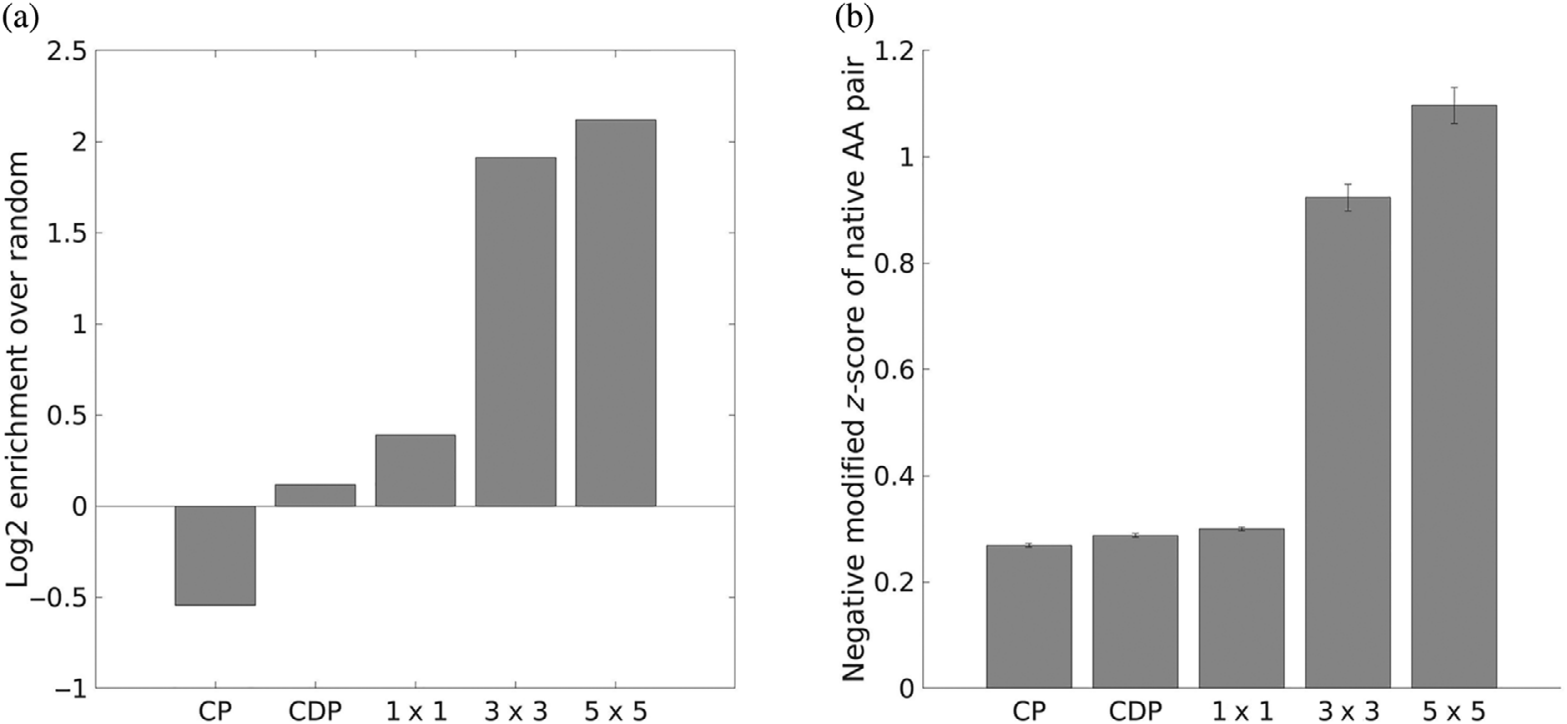
Sequence information contained in SCEs. (a) Enrichment over random choice of native amino‐acid pair identification for DB200K comparing energies for each native residue pair using a contact potential (CP), a series of contact‐degree‐dependent contact potentials (CDP), and SCEs from three types of interaction motifs, 1 × 1, 3 × 3, and 5 × 5. (b) Negative modified *z*‐score of the energies used in (a). Modified *z*‐scores were computed using medians and median absolute deviations rather than means and standard deviations. Error bars indicate the standard error of the mean

With the same set of contacts used to create the statistical potential and averaged energies in Figure 2, the SCP of each contact's interaction motif was used to predict the residue type pair of that contact. Success was measured both by identification (whether the amino‐acid pair with the most favorable pair energy is the native residue pair) and score (by how much the energy of the native residue pair differs from the energy of the most favorable pair, as measured by a modified *z*‐score; see Equation (3) in Section 4.5), both of which are shown in Figure 3. For comparison, CEs, as encoded in the aforementioned contact potential, were used to compute the same metrics. To further understand the role that structural context plays in encoding sequence preferences, the predictive performance of SCEs was computed for 1 × 1, 3 × 3, and 5 × 5 interaction motifs.

As shown in Figure 3, conditioning energies on structural context substantially improves the information they contain about native sequence preferences, with energies from 3 × 3 interaction motifs about four times as likely to identify the native residue pair as by chance (1/400), compared to the below‐chance performance of general amino‐acid pair preferences (contact potential). The poor sequence‐identification performance of the contact potential occurs because it predicts every native pair to be the most favorable one, cysteine‐cysteine (Cys‐Cys), while in actuality Cys‐Cys pairs make up less than 1/400th of the total pairs (~0.17%). Cystines are rare but very frequently occur in pairs (as either disulfide bonds or in functional sites), and for this reason the Cys‐Cys pair gets an anomalously favorable contact potential as the most over‐represented pair over expectation. In so far as statistical potentials represent interaction strength, this is not entirely wrong—Cys‐Cys pairs can form covalent bonds, which are much stronger than non‐covalent interactions of other amino‐acid pairs. However, it is not the case that disulfide bonds can be made across any proximal residue pair, and in fact strict geometric requirements have been identified for Cys‐Cys bond formation.^40^ The traditional contact potential has no choice but average out the effect of Cys‐Cys contacts across all geometric circumstances, yielding still a very strong effective contact energy. On the other hand, the SCP is able to discern where disulfides are likely and better apportion the high favorability of Cys‐Cys pairs to just the relevant geometric contexts. In fact, for 1 × 1, 3 × 3, and 5 × 5 motifs, Cys‐Cys is predicted as the most favorable contact in 36.9%, 4.3%, and 2.6% of pairs, respectively, a progressive narrowing of the geometries considered permissible for Cys‐Cys as additional structural context is incorporated.

Given that the contact potential always predicts the same amino‐acid pair to be the most favorable, the same identification and scoring tasks were tested using a series of contact‐strength‐dependent potentials so that each residue type pair was predicted based on a one‐dimensional projection of its contact geometry, termed “contact degree” (see Section 4.1). The same set of contacts used to construct the original potential was divided into 11 bins based on contact degree and a contact potential was then constructed for the set of contacts in each bin (the binning scheme used was the same as the one used to sample contacts for DB200K; see Section 4.2). To predict the amino‐acid identities of each pair, its contact degree was mapped to the corresponding bin and the contact potential for that bin was used to predict the most favorable amino‐acid pair. This serves as a better reference for the performance of the SCP, as it tests a potential that incorporates some information about contact geometry without encoding all of it as the SCP does. However, as can be seen in Figure 3, the performance (labeled “CDP”) is only marginally better than that of the contact potential, suggesting that the full structural context provides information that is difficult to encode in a single metric.

The contrast in performance between 1 × 1 and 3 × 3 motifs (Figure 3) highlights the effect of incorporating structural context further. Because 1 × 1 motifs lack flanking residues, the structural similarity of the ensembles used to calculate their energies lacks information about and therefore conflates a variety of structural contexts; a 1 × 1 ensemble implicitly constrains simple features like overall inter‐residue and relative orientation but not how the surrounding structure frames the contact. In contrast, the extensive context of 5 × 5 motifs leads to a distribution of native energies that is reliably more favorable than the median energy for each pair, likely because the choice of amino acid types in such specific contexts is even more constrained than for 3 × 3 motifs.

### 2.4 Energies conditioned on structure better correlate with experimental coupling energies

Another way to test the SCP is to evaluate how well its pair energies correspond to experimentally determined preferences. Experimental measurements that focus on pair interaction strength (i.e., the second‐order effect) would be most useful. Detailed thermodynamic measurements or high‐throughput deep mutational scans usually focus on point mutations.^41^ For instance, while ProThermDB^42^ contains measurements of how point or double mutations change the stability of their structure, there are no position pairs with measurements for more than a few residue pair combinations. However, some systems have been well studied using the double‐mutant coupling energy approach,^43^ which attempts to isolate solely the second‐order effect on stability. For example, Vinson and co‐workers have produced high‐quality coupling energy measurements for ~100 amino‐acid pairs at two inter‐chain site pairs for the dimeric parallel coiled coil system (a set of 81 for *a*–*a*′ core interactions^44^ and 16 for interfacial *g*–*e*′ interactions^45^). These coupling energies measure the change in the free energy of folding when a pair of interacting residues is simultaneously mutated to alanine, relative to when each is individually mutated to alanine.^45^ Because folding and dimerization are concomitant in this system, these coupling energies cleanly isolate just the contribution of the residues interacting in the folded state (as these interactions are absent in the unfolded/dissociated state). This an ideal scenario for comparing to SCEs, which report on the relative second‐order preferences for different amino‐acid pairs in a specific structural context.

**FIGURE 4:**
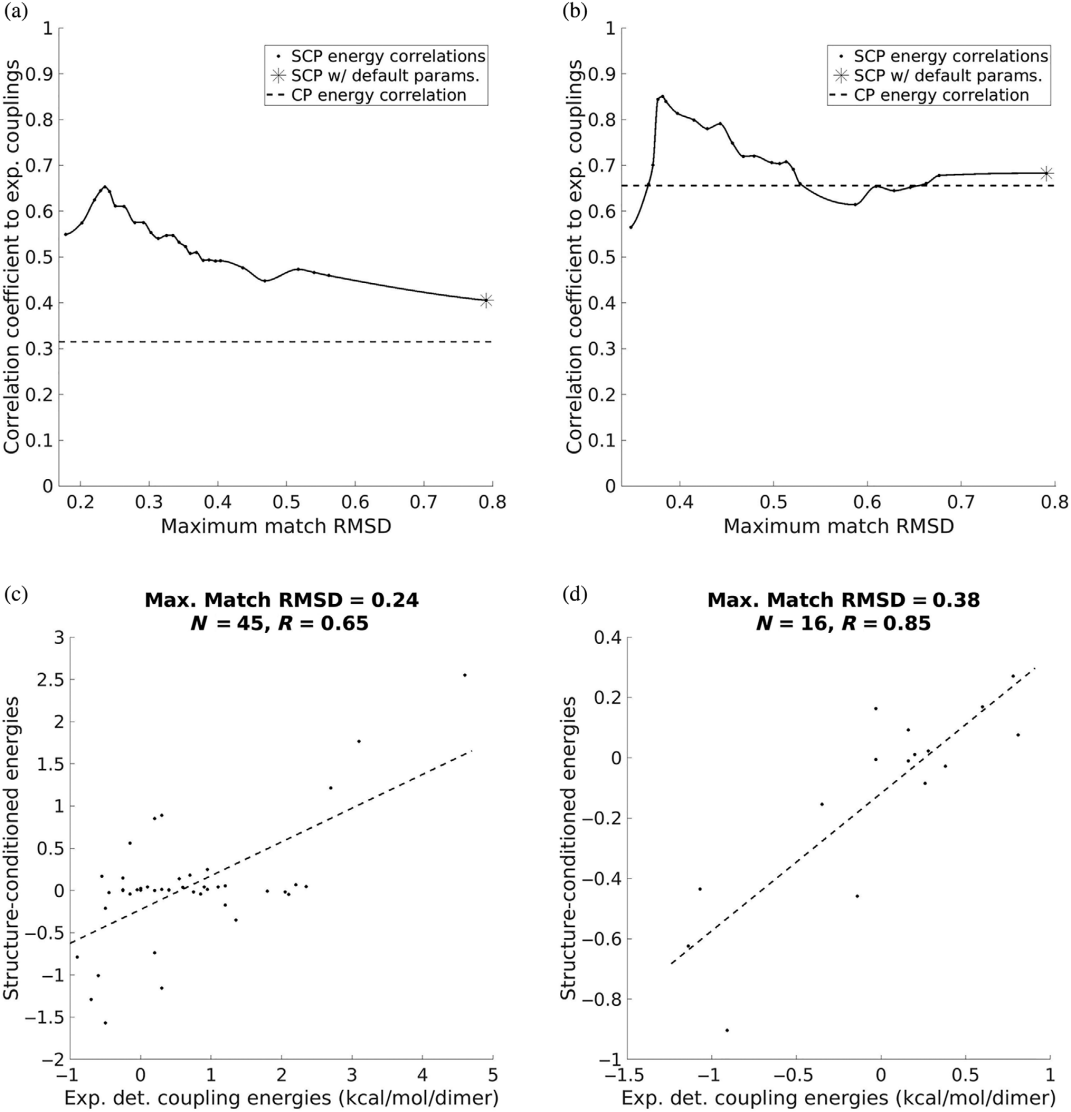
SCEs versus experimentally determined coupling energies. (a and b) Correlation between experimentally determined energies versus SCEs over a range of ensembles for *a*–*a*′ (a) and *g*–*e*′ (b) interactions. The dotted line in each plot indicates the correlation achieved by contact potential energies. The right‐most points of each plot, labeled with an asterisk (*), correspond to the energies using the default parameters. The curve in between points was computed using the “pchip” function of MATLAB and is for visualization purposes only. (c and d) Correlation between experimentally determined energies versus optimal SCEs for *a*–*a*′ (c) and *g*–*e*′ (d) interactions. The dotted line indicates the best linear fit of the data

The advantage of SCEs for capturing coupling energies is that their underlying statistics come from an ensemble of similar interaction motifs. In fact, the specific ensemble represented by the native structure would be most appropriate to use when trying to estimate true coupling energies. Of course, we do not know the native ensemble or even what RMSD neighborhood around the native interaction motif would be most appropriate to represent it. For this reason, we chose to consider multiple ensemble sizes (corresponding to a range of RMSD cutoffs) to investigate the impact on the correspondence between SCEs and experimentally determined coupling energies. Results are shown in Figure 4a,b for *a*–*a*′ and *g*–*e*′ interactions, respectively, with the performance of CEs shown with a dashed line. Interestingly, the correlation for both the *a*–*a*′ and *g*–*e*′ interactions is maximized when the ensemble is very tight (maximum RMSDs of 0.24 and 0.38, respectively), suggesting that the coiled‐coil system used to measure these coupling energies may occupy a relatively narrow native ensemble at equilibrium. But while the maximum correlations occur at low RMSDs, the correlation remains high over a broad range. For the *a*–*a*′ interactions, the correlation exceeds that of the contact potential no matter the size of the ensemble. For the *g*–*e*′ interactions, while the correlation is about equal to the contact potential's for larger ensembles, it is much higher for small ensembles except when the statistics are sparse enough that the number of matches from which SCEs are derived becomes considerably lower than the number of amino‐acid pairs (left‐most point in Figure 4b). Note that our “default” setting for computing SCEs in this work is to require a minimum ensemble of 1,000 matches to ensure that data sparsity does not come into play (see Section 4.3). The correlations for the energies computed with these default settings are shown with asterisks in Figure 4a,b and in both cases, the correlation using SCEs is higher than when using CEs.

Figure 4c,d compare experimental coupling energies to SCEs whose ensembles achieved maximal correlation for the *a*–*a*′ and *g*–*e*′ interactions, respectively. This is a striking contrast to the correlations produced by using contact potential energies (*R* = 0.65 vs. *R* = 0.31 for *a*–*a*′ energies and *R* = 0.85 vs. *R* = 0.66 for *g*–*e*′ energies; see Figure S2a,b for plots of CEs vs. experimental energies). It is clear that amino‐acid statistics from structural ensembles resembling native interaction geometries give better insights into the thermodynamic coupling between positions than the more generic preferences of a contact potential do.

### 2.5 Similar SCE patterns correspond to similar structural motifs and vice versa

Given that the amino‐acid pair statistics the SCP uses to compute its energies come from an ensemble of motifs structurally similar to the input motif, we expect pairs of structurally similar input motifs to generate similar energies. We next ask if the reverse is true, whether pairs of motifs with similar SCE matrices are structurally similar as well. The additional information about native sequence preferences contained in these energies relative to those of the contact potential (see Figure 3) suggests this may be the case, as this information gain is likely driven by the distinct amino‐acid pair statistics imposed by particular contact geometries. This potential relationship—the mutual information between contact geometry and SCEs—can be examined by clustering a large set of interaction motifs by both their structures and SCE matrices and examining the structural and energetic similarities within the resulting clusters. Taking a random subset of 50,000 motifs from DB200K, motifs were clustered by both structure (via RMSD) and energy (via *r*_E_, a function of the linear correlation of the energies as 400‐vectors; see Section 4.7). Clustering was done greedily, with clusters defined by their radius (in RMSD space for structures, and in correlation space for energies; see Section 4.7).

**FIGURE 5:**
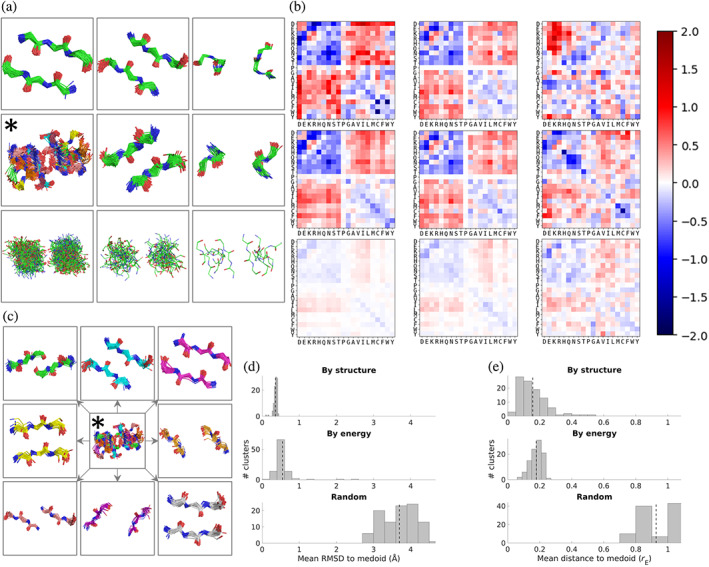
Structurally similar motifs have similar SCEs and vice versa. (a) Fragment ensembles of the top three clusters by RMSD when clustering by structure (top row), by energy (middle row), and randomly (bottom row). (b) Mean SCE matrices for the clusters shown in (a). (c) The subclusters of the cluster marked by the asterisk (*). The subclusters are sorted by size, descending, starting with the top left and going clockwise. (d and e) Distributions of RMSD and energetic similarity (*r*_E_) over the first 100 greedily obtained clusters when clustering by structure, clustering by energy, and by random assignment. For each distribution shown, the vertical dotted line indicates the mean value. Fragments were visualized with PyMOL

Figure 5 shows a summary of these clustering results. Figure 5a shows visualizations of the structures of three representative clusters in each case, Figure 5b shows the corresponding mean SCE matrices, and Figure 5d,e display the distributions of RMSDs and energetic distances (*r*_E_) over each case's first 100 clusters. Remarkably, the structural similarity of motifs clustered by energy is nearly as high as when clustered by structure; similarly, the energetic similarity of motifs clustered by structure is nearly as high as when clustered by energy. More quantitatively, the mean RMSD to the medoid (cluster representative) for the first 100 clusters is 0.36 Å when clustering by structure and 0.55 Å when clustering by energy, compared to 3.70 Å when clusters were assigned randomly (with the sizes of the random clusters chosen to match the sizes when clustering by structure). Moreover, the mean energetic distance to the medoid for the first 100 clusters is 0.16 when clustering by structure and 0.18 when clustering by energy, compared to 0.93 when clusters were assigned randomly (with the sizes of the random clusters chosen to match the sizes when clustering by energy), which is close to the *r*_E_ = 1.0 value corresponding to uncorrelated matrices. Note that in all three cases, the number of motifs being clustered is approximately the same in order to ensure the comparison is fair. As can be seen, SCE matrices contain sufficient information about the structures they were derived from that similar SCE matrices usually correspond to similar structures. Even in the cases in which the structures of SCE‐based clusters are not as mutually similar as in structure‐based clusters, the clusters are far more similar than expected by chance, with higher RMSDs usually indicative of multiple sub clusters rather than unrelated motifs, and consistent with the idea that distinct interaction geometries can induce similar energetic preferences. An example of this is shown in Figure 5c, which splits the energy‐based cluster marked by the asterisk into eight subclusters (by visual analysis in PyMOL), revealing a set of particular beta‐sheet geometries which evidently all share similar energetic preferences (see Figure S3 for additional visualizations of clusters).

**FIGURE 6:**
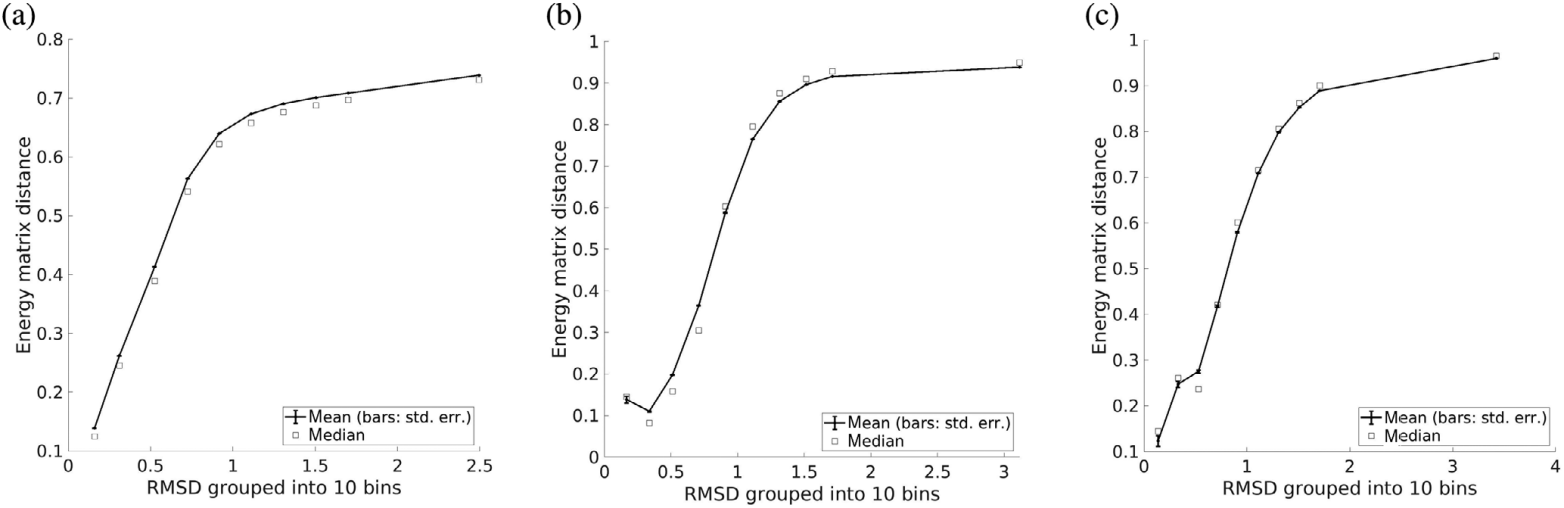
Relationship between structural similarity and energetic similarity. (a–c) 1 × 1, 3 × 3, and 5 × 5 motifs, respectively. Circles represent the mean, squares the median, and error bars the standard error

To look in more detail at how the similarity between structure and energy behaves and where it diverges, we considered a large number of pairs of interaction motifs and computed both their structural similarity (via RMSD) and energetic similarity (via *r*_E_). Specifically, the RMSD and r_E_ between each pair in a set of 20,000 motifs was computed, the pairs were partitioned into bins based on RMSD, and the average r_E_ for each bin was calculated (Figure 6). While there is some noise in the relationship, there is a clear pattern of structural similarity implying energetic similarity, with pairs of 3 × 3 motifs with an RMSD in the range [0, 0.2) having an average r_E_ of 0.14, in contrast to pairs with an RMSD in the range [1.8, 4) having an average r_E_ of 0.94, which is about what would be expected for unrelated motifs. Furthermore, an inter‐motif RMSD value of ~1.0 Å appears to be roughly where structural and energetic similarity diverge. That is, motif pairs that are closer to each other than 1.0 Å RMSD tend to exhibit various degrees of similarity in their energetic preferences in a way that strongly correlates with structural similarity, while beyond 1.0 Å energetic preferences tend to be mostly unrelated and in a way that does not strongly depend on the specific structural distance.

### 2.6 SCEs outperform traditional contact energies in native structure discrimination

**FIGURE 7:**
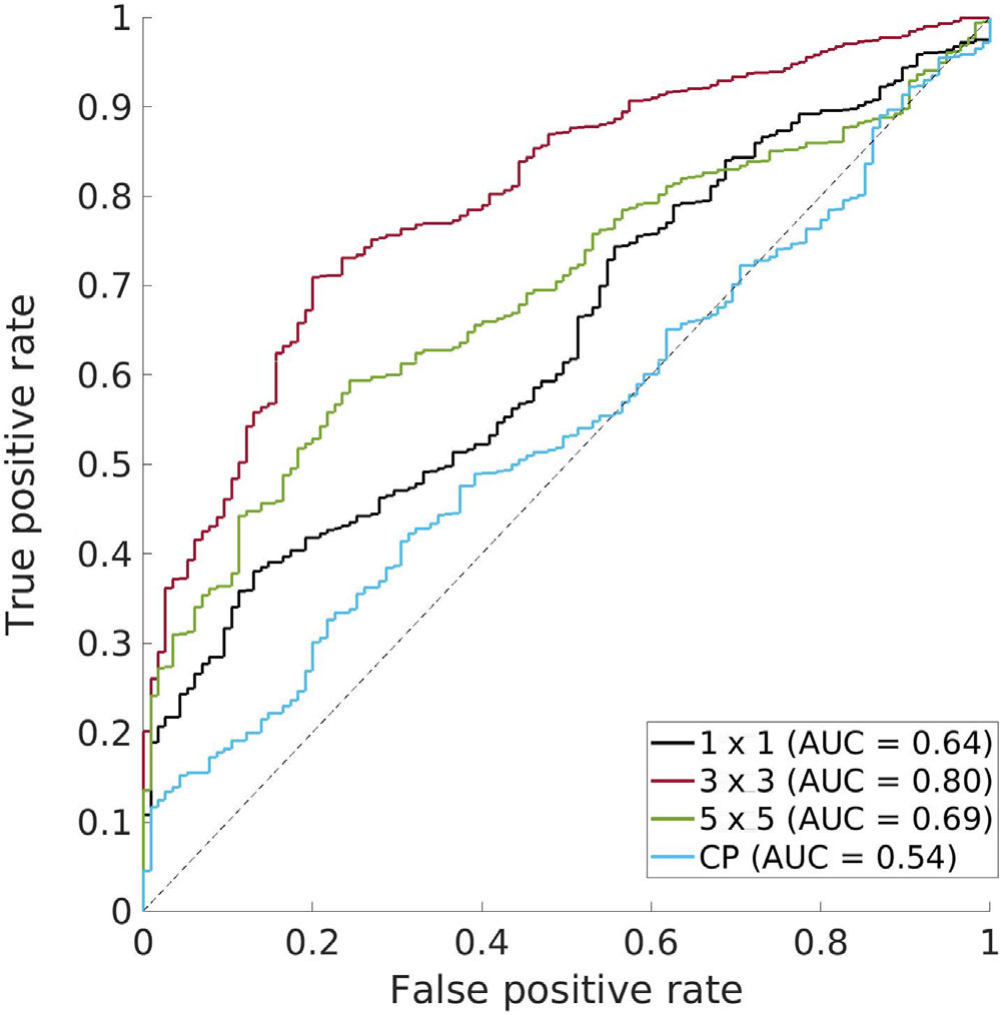
The performance of SCE‐based and CE‐based scoring functions when differentiating between low‐ and high‐quality CASP models. Each scoring function's ROC curve plots the false positive rate versus the true positive rate achieved when predicting whether or not each structure in the set of CASP models has a GDT_TS of at least 50. 1 × 1, 3 × 3, and 5 × 5 refer to the SCE‐based scoring functions using the respective motif sizes and CP refers to the CE‐based scoring function

If SCE matrices reflect how much each interaction motif prefers each possible amino‐acid pair, then scoring a structural model by the SCEs of its interacting residues should reflect how compatible the structure is with its sequence. While additional terms would be needed to fully measure sequence–structure compatibility (e.g., self‐energies as well as the pairwise SCEs), the pair energies should be informative enough to identify native‐like interactions. To test this hypothesis, data from previous Critical Assessment of protein Structure Prediction (CASP) competitions, in particular from the refinement challenges,^46^, ^47^, ^48^, ^49^, ^50^, ^51^ were collected, resulting in a large set of structures, both native and submitted models—134 targets and ~2,500 structures. For each of the 134 refinement targets from CASP9‐14, the native structure and 20 models submitted under the refinement category were scored by calculating the mean SCE of the native amino‐acid pair over all contacts (using 1 × 1, 3 × 3, and 5 × 5 motifs). As a control, each of these contacts was also scored using CEs and averaged. Following the convention of CASP, GDT_TS^52^ was used to measure the quality of each model, and this structure quality was compared to the mean SCE or CE per structure, plotting ROC curves for several GDT_TS thresholds. These curves plot the false positive rate versus the true positive rate and indicate how well each scoring function can differentiate between models higher versus lower quality models. Figure 7 shows the curve for differentiating models with a GDT_TS of at least 50 versus those with a GDT_TS less than 50. Predictive success can be measured with the area under the curve (AUC), which quantifies how likely a scoring function is to correctly rank models and ranges from 0.5 (the expected AUC for a random classifier) to 1.0 (achieved by perfectly ranking the models). The AUCs when models are scored with the mean SCE using 1 × 1, 3 × 3, and 5 × 5 motifs is 0.64, 0.80, and 0.69, respectively. In contrast, the AUC when models are scored with the mean CE is 0.54, indicating the mean CE essentially ranks models randomly with respect to GDT_TS. The increased AUCs achieved by SCE‐based scores holds when using other thresholds of GDT_TS (60, 70, 80, 90; see Figure S4).

The substantially larger AUCs achieved by the SCE‐based scores, in particular the 0.8 achieved using 3 × 3 motifs, is another piece of evidence for the SCP encoding additional information about native sequence preferences compared to the CP and shows this information can be exploited to evaluate the structural quality of predicted models. The improved predictions using the 3 × 3‐based scores over the 1 × 1‐based scores is expected, but it is less clear why there is a lack of systematic improvement by the 5 × 5‐based scores. It may result from limitations associated with the sparser and less robust statistics of larger structural motifs. To see whether the relationship between SCE‐based scores and GDT_TS generalizes to other measures of structural quality, the same experiment was performed using TM‐score^53^ (Figure S5), another common measure of structure quality, and RMSD (Figure S6). In both cases, the same pattern was observed, with SCEs predicting structural quality more effectively than CEs across all tested thresholds of TM‐score and RMSD.

## 3 DISCUSSION

The long history and widespread use of contact potentials suggests that extensions to the concept may also prove fruitful. Here, we demonstrated such an extension and showed that conditioning the contact potential's amino‐acid pair statistics on the backbone conformation of the interacting fragment is a promising way to increase the relevance of the resultant statistical energies. Moreover, examining these structure‐conditioned energies and the fragment geometries they are conditioned on has revealed a general relationship between structural similarity and energetic similarity.

The results summarized in Figure 3 provide a good example of this increase in relevance, highlighting how the energies of the SCP contain more native sequence information than those of a traditional CP. Indeed, while the CP effectively encodes common patterns of amino‐acid pair interactions—the most favorable energies after Cys‐Cys being Glu‐Lys, Asp‐Lys, Arg‐Asp, and Arg‐Glu (all complementarily‐charged amino‐acid pairs which frequently interact)—it has no mechanism to recognize any detailed structural context in which these interactions occur. This can be seen as a limitation of CPs that is accepted in exchange for speed. Thus, a CP serves as a fast but very approximate measure of a structure's inter‐residue interactions.

In contrast, the SCP described here performs much better. Importantly, one would not expect a contact potential of any kind, which inherently captures second‐order sequence preferences only, to have a high sequence recovery. In fact, the most significant explanatory effect of sequence is expected to reside in the first‐order contribution (e.g., preference for degree of burial, backbone dihedral angles, etc.). Further, when it comes to second‐order effects, it is the sum of pair interactions involving a given residue that most matters for amino‐acid choice at the position, while each pair contribution may play only a minor role. Despite this, the SCP stills exhibits considerable predictive performance in picking out native amino‐acid pairs, with a rate of four times over that expected by chance. In terms of speed, the general relationship between structural similarity and energetic similarity shown in Figure 6 implies that SCE matrices do not need to be computed for every fragment of interest. Instead, a database of energies can be pre‐computed, and the interaction motifs of interest can be evaluated on the fly by looking up the energies of the most similar motifs in the database. This would make the speed of SCPs comparable to that of CPs, while providing a considerably more accurate sequence–structure linkage. A relevant comparison is the residue‐pair‐transform (RPX) construct in Rosetta.^54^ While this method scores residue pairs using the Rosetta energy function, not structural statistics as the SCP does, and uses spatial binning as the measure of structural similarity, not RMSD, both models account for how interaction geometry affects sequence compatibility by examining sets of spatially similar contacts in order to more accurately evaluate pairs of interacting residues.

Additional evidence of the relevance of SCP‐based energies is shown in Figure 4, which demonstrates that SCE matrices are more closely related to thermodynamic coupling energies in coiled coils. While SCEs correlate more highly with experimental coupling values than CEs do using the “default” parameters, the highest correlations occur when the ensemble of motifs used to condition the amino‐acid pair statistics were constrained to include only those fragments with very high structural similarity to the query motifs (i.e., the motifs centered on the *a*–*a*′ and *g*–*e*′ interaction pairs). This sensitivity to the chosen ensemble reinforces the point that structural context matters when estimating pairwise energies and suggests that using statistical energies as proxies for coupling energies requires knowledge of the native ensemble, not just knowledge of the crystallized conformation. While such knowledge would be rarely available, the SCP at least provides a means of tuning the statistics based on the predicted or assumed ensemble, which cannot be done with a CP. Measurements such as B‐factors might provide insight into this, although the availability of additional experimental coupling measurements would be required to determine the relationship between them. Moreover, in some cases such as sequence design, it may be useful to impose a desired ensemble, making this tuning capability of the SCP convenient. Considering the results in Figure 4 more broadly, it is interesting that the statistics in the PDB, when conditioned on an appropriate set of fragments, correspond even approximately with coupling energies, which are derived from measurements of thermodynamic equilibria in specific systems. This correspondence suggests that thermodynamic preferences contribute to the distribution of amino‐acid pair statistics in the PDB.

Results in Figures 7 and S4–6 corroborate those in Figures 3 and 4 by directly evaluating how effective each energy function is at predicting the sequence–structure compatibility in structural models. The increase in AUCs (which quantify how well high‐quality structures can be differentiated from low‐quality ones) achieved by SCEs shows that incorporation of structural context helps in the evaluation of pairwise interactions in structural models. Importantly, the dataset involved in this analysis is expansive, comprising thousands of structural models submitted by CASP participants, and includes models derived from a variety of techniques tried over many years. The consistent increase in AUCs, whether using GDT_TS, TM‐score, or RMSD, and regardless of the threshold chosen to split high‐quality and low‐quality models, allows us to conclude that an SCP‐based scoring function is reliably better than an equivalent CP‐based one. This holds even when the SCEs are computed with the minimally contextual 1 × 1 motifs, but the further increase when using 3 × 3 motifs confirms that the increase in performance is driven by incorporating additional structural context.

Perhaps the most striking observation about the SCP is the bidirectional relationship it reveals between contact geometry and pairwise sequence preferences, which is shown via clustering in Figure 5 and more generally in Figure 6. It may be expected that structurally similar pairs of interaction motifs induce similar SCE matrices. After all, these matrices are computed by collecting ensembles of structurally similar fragments and pooling their amino‐acid pair statistics. However, it is interesting that the relationship holds in the other direction—that energetically similar pairs of motifs tend to be structurally similar as well. It could have been the case that most contact geometries would induce similar SCE matrices, which would have precluded using energetic similarity to predict structural similarity. This is not the case, however, and Figure 5d makes it clear that structurally unrelated motifs are very unlikely to have similar SCE matrices. This coupling between structural and energetic similarity, the limits of which are quantified in Figure 6, implies that not only does a particular contact geometry impose constraints on sequence preferences, but that particular sequence preferences impose constraints on the contact geometry. In effect, there appears to (usually) be at most only one way, in local structural space, to achieve a specific set of pair amino‐acid preferences.

At a high level, our results show that local geometry‐to‐sequence mappings are inherently learnable, generalizable, and with a tight coupling between local structure and the pattern of amino‐acid preferences. This fact may well be a part of the reason behind recent results having been able to achieve excellent generalization capabilities in going from multiple‐sequence alignments to accurately predicted structures.^37^, ^38^ These results once again suggest that the amount of protein structural data amassed to date is sufficient to establish robust generalizations about sequence–structure relationships. Thus, continued exploitation of these data is likely to produce additional insights and generalizations and may enable novel techniques for the design and modeling protein structure and properties.

## 4 MATERIALS AND METHODS

### 4.1 Contact degree

Contacts in this study were defined using our previously described contact degree (CD) metric.^21^, ^55^, ^56^ CD quantifies the extent to which a pair of positions is poised to host a contact by placing all possible rotamers (of all natural amino acids) at both positions and calculating the probability‐weighted fraction of mutually exclusive rotamer pairs (i.e., those with clashing heavy atoms; see Holland et al.^56^).

In more detail, the contact degree between two residue positions *p*_*i*_ and *p*_*j*_ is defined as follows. The Dunbrack backbone‐dependent rotamer library^57^ is used to determine the set “allowed” rotamers at each position (i.e., rotamers with strong clashes with the main chain) and their probabilities of occurrence. For each pair of allowed rotamers *r*_*i*_ and *r*_*j*_ (at *p*_*i*_ and *p*_*j*_, respectively) the distance between each pair of heavy (non‐hydrogen) atoms is calculated. If any pair of heavy atoms between *r*_*i*_ and *r*_*j*_ is within 3 Å, *r*_*i*_ and *r*_*j*_ are considered mutually interfering. The contact degree between *p*_*i*_ and *p*_*j*_ is the probability‐weighted fraction of interfering rotamer pairs out of all placeable ones:

$$\begin{array}{rcl} \mathrm{CD}\left(p_{i},p_{j}\right) & = & \sum_{a=1}^{20}\sum_{b=1}^{20}\sum_{r_{i}\in R_{i}\left(a\right)}\sum_{r_{i}\in R_{i}\left(b\right)}C\left(r_{i},r_{j}\right)\cdot \mathbb{P}\left(a\right)\\ & & \cdot \mathbb{P}\left(b\right)\cdot \mathbb{P}\left(r_{i}\right)\cdot \mathbb{P}\left(r_{j}\right). \end{array}$$

Above, *R*_*i*_(*a*) is the set of rotamers of amino acid *a* allowed at position *p*_*i*_, *C*(*r*_*i*_, *r*_*j*_) is 1 if *r*_*i*_ and *r*_*j*_ are interfering and 0 otherwise, ℙ(*a*) is the probability of amino acid *a*, and ℙ(*r*_*i*_) is the probability of rotamer *r*_*i*_ according to the backbone‐dependent rotamer library. Note that a CD of 0 indicates no pairs of placeable rotamers are clashing (no contact) and a CD of 1 indicates all pairs are (strongest contact).

### 4.2 Contact database creation

In order to sample a diverse distribution of native contacts, a high quality, nonredundant subset of the PDB was collected and around 200,000 contacts were sampled from it. In more detail, the PISCES server^58^ was used to collect a nonredundant subset of the PDB. Only structures solved by X‐ray crystallography were included, with the maximum resolution capped at 2.3 Å and the maximum *R*‐value at 0.3. Structures were filtered by chain, keeping only those with between 40 and 10,000 residues, and with the maximum sequence identity of any pair restricted to 25%. The list of chains meeting these criteria was collected on January 1, 2020, and resulted in 12,148 entries. Contacts were computed between every pair of residues in every chain. In order to sample inter‐protein contacts, contacts were also computed between every pair of residues between each chain and every other chain in its PDB file. This resulted in 61,510,642 contacts in total. Contacts were then filtered to include only those for which both contacting residues had canonical amino acid names (with MSE being considered equivalent to MET), and enough sequence separation (at least five residues in between the two contacting ones) to ensure the largest considered interaction motifs (5 × 5‐mers) were composed of two non‐contiguous segments. Furthermore, a contact was included only if each residue involved in the 5 × 5‐mer motif had all four backbone atoms. Because most contacts are weak, and we wanted to sample many strong contacts in addition to weak ones, the contact database was created by sampling an equal number of contacts from 11 bins of contact degree: [0, 2^−10^), [2^−10^, 2^−9^), [2^−9^, 2^−8^), …, [2^−1^, 1]. DB200K was created by sampling 18,182 contacts per bin, resulting in 200,002 contacts spanning 10,837 structures/complexes. The set of contacts comprising DB200K can be found in the “DB200K.tar.gz” file hosted on Zenodo.^59^

### 4.3 Structure‐conditioned potentials

An interaction motif is a structural fragment centered around a pair of contacting residues. The fragment may contain just the pair of interacting residues (1 × 1) or one or more flanking residues on each side of the pair (3 × 3, 5 × 5, etc.). Corresponding to the pair component of the dTERMen energy function, the structure‐conditioned energy (SCE) matrix for an interaction motif is a 20 × 20 matrix containing log‐transformed ratios of amino‐acid pair observations over expectations, computed using the amino‐acid statistics from an ensemble of fragments structurally similar to the interaction motif. Only the statistics of the two contacting residues are considered; the flanking residues control how much structural context is considered when collecting the ensemble of similar fragments, but their amino‐acid identities do not contribute directly to the energies. The ensemble is collected by using the interaction motif to query into a structural database, finding all fragments (matches) of the same size as the query in the order of their backbone‐atom root‐mean‐square deviation (RMSD) from the query motif (all four backbone atoms, N, Cα, C, and O, are included in this calculation). The search is limited by both the number of fragments returned (max count) and the maximal RMSD to the query (RMSD cutoff). Except for the SCEs used to compare with experimental coupling energies, which were recomputed using a wide variety of match counts (see Figure 4 and Section 4.6), the max count was fixed at 50,000 for all SCEs computed here. The RMSD cutoff was set in a size‐based manner according to our previously derived cutoff function (eqs. 25 and 26 in the supplementary information of Zhou et al.^21^). This resulted in cutoffs of 1.0, ~0.79, and ~ 0.77 Å for 1 × 1, 3 × 3, and 5 × 5 motifs, respectively.

The equation below details how the 400 structure‐conditioned energies (SCEs) of an SCP are computed for an interaction motif *f* centered around a pair of interacting positions (*i*, *j*):

$$\mathrm{SCE}\left(a_{i},a_{j}\right)=-\log\frac{N_{\mathrm{obs}}\left(a_{i},a_{j}\right)+\varepsilon }{N_{\exp}\left(a_{i},a_{j}\right)+\varepsilon }.$$

Here, (*a*_*i*_, *a*_*j*_) is the amino‐acid pair whose SCE is being computed at these positions (with *a*_*i*_ being the amino acid at position *i* and *a*_*j*_ the amino acid at position *j*), *N*_obs_(*a*_*i*_, *a*_*j*_) is the number of occurrences of pair (*a*_*i*_, *a*_*j*_) in *f*'s ensemble of matching fragments, and *N*_exp_(*a*_*i*_, *a*_*j*_) is the number of occurrences of pair (*a*_*i*_, *a*_*j*_) that would be expected if there were no pair preferences. The pseudocount *ε* is set to 20maxNobsaiajNexpaiaj1. Note the above equation is equivalent to Equation (2) but with *a* replaced by *a*_*i*_ and *b* replaced by *a*_*j*_ for clarity about how *i* and *j* play a role. *N*_exp_(*a*_*i*_, *a*_*j*_) is defined as follows:

$$N_{\exp}\left(a_{i},a_{j}\right)=\sum_{m\in \mathrm{AA}}\frac{\exp\left(-E_{1}\left(a_{i}|m_{i}\right)-\Delta_{i}\left(a_{i},M\right)\right)}{\sum_{a\in \mathrm{AA}}\exp\left(-E_{1}\left(a|m_{i}\right)-\Delta_{i}\left(a,M\right)\right)}\times \frac{\exp\left(-E_{1}\left(a_{j}|m_{j}\right)-\Delta_{j}\left(a_{j},M\right)\right)}{\sum_{a\in \mathrm{AA}}\exp\left(-E_{1}\left(a|m_{j}\right)-\Delta_{j}\left(a,M\right)\right)}.$$

Here, *m* is a match in *f*'s ensemble of matching fragments *M*, *E*_1_(*a*_*i*_ | *m*_*i*_) is the pseudo‐energy associated with amino acid *a*_*i*_ at position *i* in match *m*, *AA* is the set of the 20 natural amino acids, and Δ_*i*_(*a*_*i*_, *M*) is a residual energy associated with amino acid *a*_*i*_ that is set (for each motif) to ensure that the expected marginal counts of each amino acid at each position coincide with observed counts. The pseudo‐energy in *E*_1_ is a first‐order energy model that considers the backbone dihedral angles *φ*, *ψ*, and *ω* and environment to estimate how favorable each amino acid is at a given position. The first term under the outer sum is thus a ratio between how likely (according to *E*_1_) amino acid *a*_*i*_ is to occur at position *i* and the sum of the likelihoods over all possible amino acids at this position. The second term under the outer sum computes the same ratio but for *a*_*j*_ at position *j*. Multiplied together, these two terms capture the probability of observing pair (*a*_*i*_, *a*_*j*_) in match *m* under a model that knows about first‐order amino‐acid preferences but assumes no second‐order dependencies. This means the sum over each match in the ensemble *M* is the expected number of observations in *M* of the amino acid pair (*a*_*i*_, *a*_*j*_) if there were no second‐order (or other higher‐order) dependencies. Thus, the ratio between *N*_obs_(*a*_*i*_, *a*_*j*_) and *N*_exp_(*a*_*i*_, *a*_*j*_) estimates to what extent true observations exhibit apparent correlations.

In more detail, E_1_ is a hierarchical model estimating how much each amino acid prefers occupying regions of backbone dihedral angles and environment. First, φ/ψ‐space is divided into uniformly sized bins of 10° × 10° and the preference for each amino acid to occupy each bin is estimated with a statistical potential. That is, the number of observations of an amino acid are compared to the number expected based on its frequency in the database. Then, *ω*‐space is divided into non‐uniformly sized bins (due to regions of sparse statistics) and the preference for each amino acid to occupy each bin given its *φ*/*ψ* preferences is estimated with a conditional potential. That is, the number of observations of an amino acid are compared to the number expected given the *φ*/*ψ* preferences of these observations. Finally, the preference for each amino acid to occupy various levels of burial is estimated with a potential conditioned on the previous two. Together, these three potentials capture the self‐preferences of each amino‐acid type. The residual energy term, Δ_*i*_(*a*_*i*_, *M*), is an adjustment energy that is included to preserve the marginal amino‐acid distributions at individual coupled positions of the pair TERM. This is an approximation that works well in practice, where we assume that perturbations in the marginal distributions at individual sites of a pair motif are caused by residual first‐order effects, rather than second‐order ones. The correction term is calculated as:

$$\Delta_{i}\left(a_{i},M\right)=-\ln\left(\frac{N_{o}\left(a_{i}\right)}{N_{e}\left(a_{i}\right)}\right),$$

where $N_{o}\left(a_{i}\right)$ is the number of times amino acid $a_{i}$ is observed in the corresponding position of matching pair fragments and $N_{e}\left(a_{i}\right)$ is the number of times this amino acid was expected at this position, based on the $E_{1}$ background potential:

$$N_{e}\left(a_{i}\right)=\sum_{m\in M}\frac{\exp\left(-\left[E_{1}\left(a_{i}|m_{i}\right)\right]\right)}{\sum_{a=1}^{20}\exp\left(-\left[E_{1}\left(a|m_{i}\right)\right]\right)}.$$

See the “Pre‐computed contributions” and “Pair contributions” sections of the supplementary information of Zhou et al.^21^ for more information about pseudo‐energies and residual energies, respectively.

The structural database used to search for matches was created by compiling a nonredundant subset of the PDB on a chain‐by‐chain basis. In particular, the first chain in each cluster of BLASTclust,^60^ which clusters structures in the PDB by chain, was considered. The sequence identity between each pair of sequences was limited to at most 30% and only structures solved by X‐ray crystallography with a resolution of at most 2.6 Å were included in the database, resulting in 23,643 structures in total. BLASTclust clusters were downloaded on January 22, 2019.

### 4.4 Contact potential

The contact potential was computed for every canonical amino‐acid pair using Equation (1), with DB200K used as the set of contacts. Since by construction, the energy is invariant to the order of the amino‐acid pairs, with *E*(*a*, *b*) = *E*(*b*, *a*), the number of observed pairs *N*_obs_(*a*, *b*) must take this into account by summing (*a*, *b*) and (*b*, *a*) contacts together:

$$N_{\mathrm{obs}}\left(a,b\right)=N_{\mathrm{obs}}\left(b,a\right)=N\left(a,b\right)+\left(1-𝕀\left(a,b\right)\right)\cdot N\left(b,a\right).$$

Here, *N*(*a*, *b*) is the number of contacts in the database between *a* and *b* in the order (*a*, *b*), and 𝕀(*a*, *b*) is 1 if *a* = *b* and 0 otherwise, ensuring homotypic contacts are not counted twice. To compute the number of expected pairs *N*_exp_(*a*, *b*), the expectation for heterotypic pairs must be doubled so that, in accordance with the chosen reference state, in the absence of amino‐acid pair preferences between *a* and *b*, *N*_obs_(*a*, *b*) = *N*_exp_(*a*, *b*), thereby making *E*(*a*, *b*) = 0:

$$\begin{array}{l} N_{\exp}\left(a,b\right)=N_{\exp}\left(b,a\right)=N_{\mathrm{obs}}\left(a\right)\cdot N_{\mathrm{obs}}\left(b\right)\cdot H\left(a,b\right)/N\\ H\left(a,b\right)=2-𝕀\left(a,b\right). \end{array}$$

The empirically derived amino acid‐dependent pseudocount *ε* was structured identically to the one used for the SCP (see Section 4.3 and Zhou et al.^21^ for details), although in the case of the contact potential, the large set of contacts used to compute the statistics ensured there was no data sparsity and thus the values were negligible.

### 4.5 AA pair identification

The modified *z*‐score used in Figure 3b is computed by analogy to a traditional *z*‐score, but with the mean replaced by the median and the standard deviation replaced by the median absolute deviation (MAD). Below, *E* is the set of 400 energies in an SCP and *E*_*i*_ is the one of these energies whose modified *z*‐score is being computed:

(3) $$Z_{\mathrm{mod}}\left(E_{i}\right)=\frac{E_{i}-\text{median}\left(E\right)}{\mathrm{MAD}\left(E_{i}\right)}=\frac{E_{i}-\text{median}\left(E\right)}{\text{median}\left(\left|E_{i}-\text{median}\left(E\right)\right|\right)}.$$

### 4.6 Coupling energies

Experimentally determined coupling energies for the *a*–*a*′ and *g*–*e*′ interhelical interactions were taken from table 4 and table III from Acharya et al.^44^ and Krylov et al.,^45^ respectively. Since experimentally solved structures for the coiled coils were not available, they were modeled using CCFold^61^ based on the sequences specified in the papers, trimming off N‐ and C‐terminal residues distal to the interaction. In particular, for the system used for measuring the *a*–*a*′ interactions, the sequences used were RAAFLEKENTALRTRLAELRKRVGRCRNIVSKYETRYG (chain A) and RAAFLEKENTALRTELAELEKEVGRCENIVSKYETRYG (chain B), with the residue pair (A16, B16) used to compute the energies (note that these residues, both labeled as X in the sequence in Acharya et al.,^44^ were replaced with leucine when modeled with CCFold). For the system used for measuring the *g*–*e*′ interactions, the sequence used for both chains was KVFVPDEQKDEKYWTRRKKNNVAAKRSRDARRLKENQITIRAAFLEKENTALRTEVAELRKEVGRCKNIVSKYETRYGPL, with the residue pair (A41, B46) used to compute the energies. The CCFold structural models of both dimers are available as PDB files (see “cc‐a‐a.pdb” and “cc‐g‐e.pdb” in the Supporting Information for the structures used for the *a*–*a*′ and *g*–*e*′ systems, respectively).

### 4.7 Clustering

Each clustering was performed by randomly sampling without replacement a set *S* of 50,000 motifs from DB200K and running 100 rounds of an in‐house greedy clustering method which accepts two parameters, a distance cutoff *d* and a sampling count *n*, and returns one cluster per round. In any round *i*, *n* motifs are randomly sampled without replacement from *S*. The distance between each pair of *n* motifs is computed and the motif with the largest number of distances of at most *d* to the other sampled motifs is chosen as the cluster representative *r*. The distance between r and each motif in S is computed and every motif with a distance of at most *d* is included in the returned cluster *C*. For round *i* + 1, *S* = *S* \ *C* (i.e., the elements assigned to the *i*th cluster are not available for rounds *i* + 1, *i* + 2, …). When clustering by structure, the distance metric used was best‐fit RMSD and *d* was chosen to be 0.5. When clustering by SCE, the distance metric used was *r*_E_ = 1 – *r*, where *r* is the linear correlation coefficient between the pair of SCE matrices, and *d* was chosen to be 0.3. Note that the chosen values of *d*, 0.5 when clustering by structure and 0.3 when clustering by energy, result in the top 100 clusters including a similar number of motifs, making the comparisons of distributions in Figure 5d,e fair. To make the sets of random clusters a similarly fair control, the clusters were chosen to have the same number of motifs as the structure clusters (in Figure 5d) and the energy clusters (in Figure 5e) as well.

### 4.8 CASP model evaluation

All publicly available refinement targets from CASP9‐14 were considered, with the solved structure and up to 20 models included per target. To sample a set of structures with a wide range of structure quality, the 20 models included for each target were selected by sorting the models best‐to‐worst (by GDT_TS) and alternately adding the next‐best and next‐worst models remaining until either 20 were added or no more models were available. The set of targets and their included models is listed in the “CASP‐models.xlsx” file hosted on Zenodo.^59^ Structures and GDT_TS scores were taken from the CASP website (https://predictioncenter.org/). TM‐scores and RMSDs were computed using the TM‐score program^53^ with the default settings. SCEs were computed over every contact with a CD of at least 0.1.

## SUPPLEMENTARY MATERIAL

Supplementary figures can be found in the file “supplementary.docx.” The structures used to compute the structure‐conditioned energies of the two coiled‐coil systems are included as “cc‐a‐a.pdb” and “cc‐g‐e.pdb.” Additional supplementary material has been uploaded to Zenodo.^59^ This additional material contains two files: “CASP‐models.xlsx,” which lists the identities and GDT_TS scores of the CASP models described in Section 4.8, and “DB200K.tar.gz,” which is an archived directory containing the energy files comprising the DB200K database described in Section 4.2.

## CONFLICT OF INTEREST

The authors declare no potential conflict of interests.

## AUTHOR CONTRIBUTIONS

**Jack Edward Holland:** Conceptualization (equal); data curation (lead); formal analysis (equal); investigation (equal); methodology (equal); software (lead); visualization (lead); writing – original draft (equal); writing – review and editing (equal). **Gevorg Grigoryan:** Conceptualization (equal); formal analysis (equal); funding acquisition (lead); investigation (equal); methodology (equal); project administration (lead); software (supporting); visualization (supporting); writing – original draft (equal); writing – review and editing (equal).

## Supporting information


**Figure S1**. Additional information about correlations between SCEs and CEs. Top: Distribution of correlation coefficients between each set of SCEs in DB200K and the CEs. The dotted line indicates the mean correlation of *R* = 0.20. Middle: Bottom: Plot of the averaged, symmetrized SCEs from 1 × 1‐ (Middle) or 5 × 5‐motifs (Bottom) versus those from a contact potential. As with Figure 2a, Cys‐Cys is not shown as it occupies points to the far bottom‐left (mean SCE of −1.70 (1 × 1) or −0.88 (5 × 5)), though its inclusion increases the correlation to *R* = 0.91 (1 × 1) or *R* = 0.84 (5 × 5).
**Figure S2**. CEs versus experimentally determined coupling energies. Top, Bottom: Correlation between experimentally determined energies versus CEs for *a*–*a*′ (Top) and *g*–*e*′ (Bottom) interactions. The dotted line indicates the best linear fit of the data.
**Figure S3**. Additional clustering visualizations. The first three figures show the fragment ensembles of the top 100 clusters when clustering by structure, energy, or randomly, respectively. The bottom three figures show the mean SCE matrices for these respective clusterings. The color scale is the same as shown in Figure 5.
**Figure S4**. Relationship between GDT_TS and statistical energies over a set of predicted CASP models and their corresponding native structures via ROC curves. A–C: SCEs versus GDT_TS. (A), (B), and (C) correspond to 1 × 1, 3 × 3, and 5 × 5 SCEs. D: CEs versus GDT_TS.
**Figure S5**. Relationship between GDT_TS and statistical energies over a set of predicted CASP models and their corresponding native structures via ROC curves. A–C: SCEs versus TM‐score. (A), (B), and (C) correspond to 1 × 1, 3 × 3, and 5 × 5 SCEs. D: CEs versus TM‐score.
**Figure S6**. Relationship between GDT_TS and statistical energies over a set of predicted CASP models and their corresponding native structures via ROC curves. A–C: SCEs versus RMSD. (A), (B), and (C) correspond to 1 × 1, 3 × 3, and 5 × 5 SCEs. D: CEs versus RMSD.

**Data S1**. Supporting Information (cc‐a‐a.pdb).

**Data S2**. Supporting Information (cc‐g‐e.pdb).
