# Supplementary material for: Structure‐conditioned amino‐acid couplings: How contact geometry affects pairwise sequence preferences
Source: Protein Sci. 2022 Feb 15;31(4):900–17. doi: 10.1002/pro.4280 (PMC8927866; doi:10.1002/pro.4280)
Supplement: Supplementary file 1 — Figure S1. Additional information about correlations between SCEs and CEs. Top: Distribution of correlation coefficients between each set of SCEs in DB200K and the CEs. The dotted line indicates the mean correlation of R = 0.20. Middle: Bottom: Plot of the averaged, symmetrized SCEs from 1 × 1‐ (Middle) or 5 × 5‐motifs (Bottom) versus those from a contact potential. As with Figure 2a, Cys‐Cys is not shown as it occupies points to the far bottom‐left (mean SCE of −1.70 (1 × 1) or −0.88 (5 × 5)), though its inclusion increases the correlation to R = 0.91 (1 × 1) or R = 0.84 (5 × 5). Figure S2. CEs versus experimentally determined coupling energies. Top, Bottom: Correlation between experimentally determined energies versus CEs for a–a′ (Top) and g–e′ (Bottom) interactions. The dotted line indicates the best linear fit of the data. Figure S3. Additional clustering visualizations. The first three figures show the fragment ensembles of the top 100 clusters when clustering by structure, energy, or randomly, respectively. The bottom three figures show the mean SCE matrices for these respective clusterings. The color scale is the same as shown in Figure 5. Figure S4. Relationship between GDT_TS and statistical energies over a set of predicted CASP models and their corresponding native structures via ROC curves. A–C: SCEs versus GDT_TS. (A), (B), and (C) correspond to 1 × 1, 3 × 3, and 5 × 5 SCEs. D: CEs versus GDT_TS. Figure S5. Relationship between GDT_TS and statistical energies over a set of predicted CASP models and their corresponding native structures via ROC curves. A–C: SCEs versus TM‐score. (A), (B), and (C) correspond to 1 × 1, 3 × 3, and 5 × 5 SCEs. D: CEs versus TM‐score. Figure S6. Relationship between GDT_TS and statistical energies over a set of predicted CASP models and their corresponding native structures via ROC curves. A–C: SCEs versus RMSD. (A), (B), and (C) correspond to 1 × 1, 3 × 3, and 5 × 5 SCEs. D: CEs versus RMSD. [file PRO-31--s003.docx]

Supplementary Figures for Structure-conditioned amino-acid couplings: how contact geometry affects pairwise sequence preferences

Jack Holland^1^, Gevorg Grigoryan^1*^

^1^Department of Computer Science, Dartmouth College, Hanover, NH 03755, USA

*Corresponding author: [gevorg.grigoryan@dartmouth.edu](mailto:gevorg.grigoryan@dartmouth.edu)


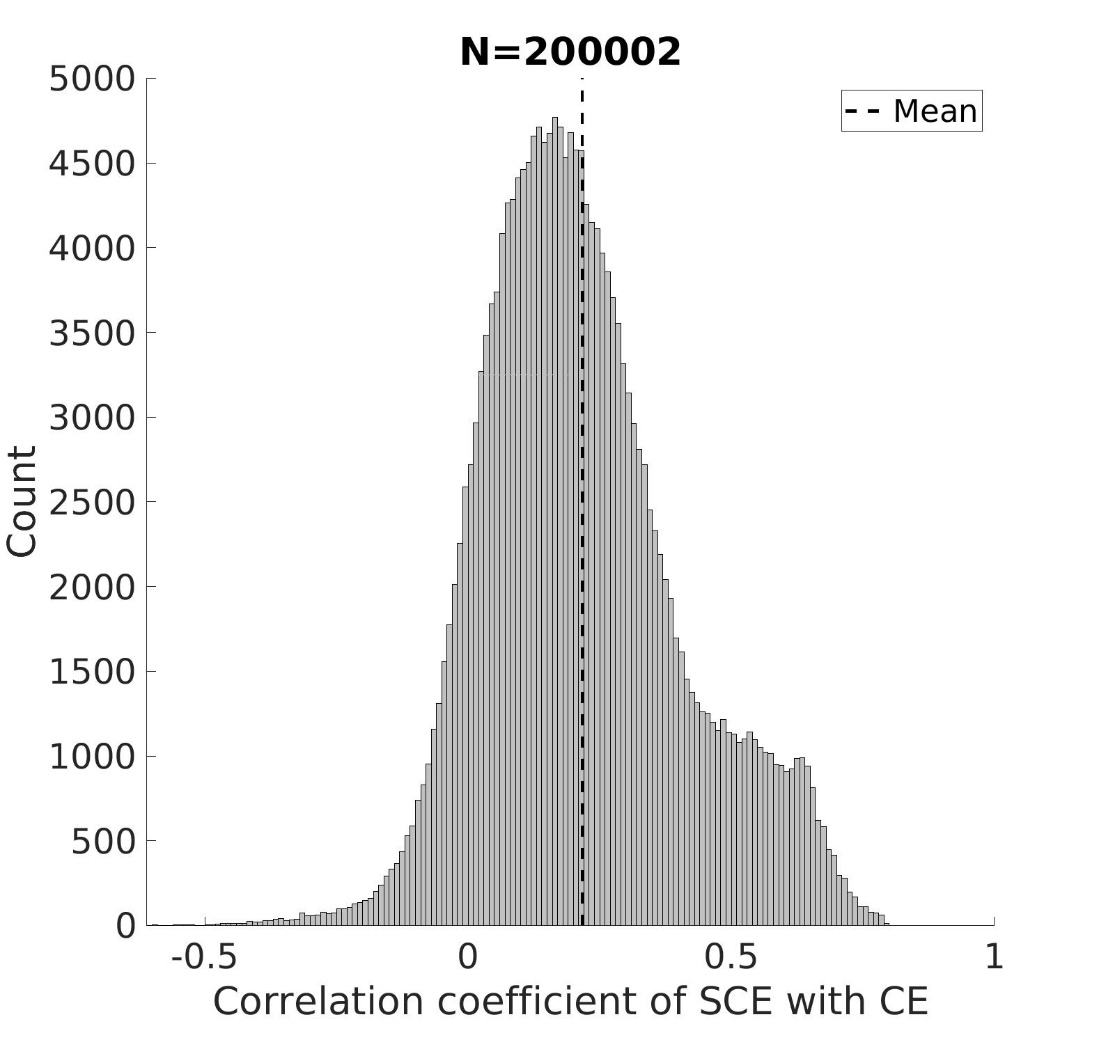


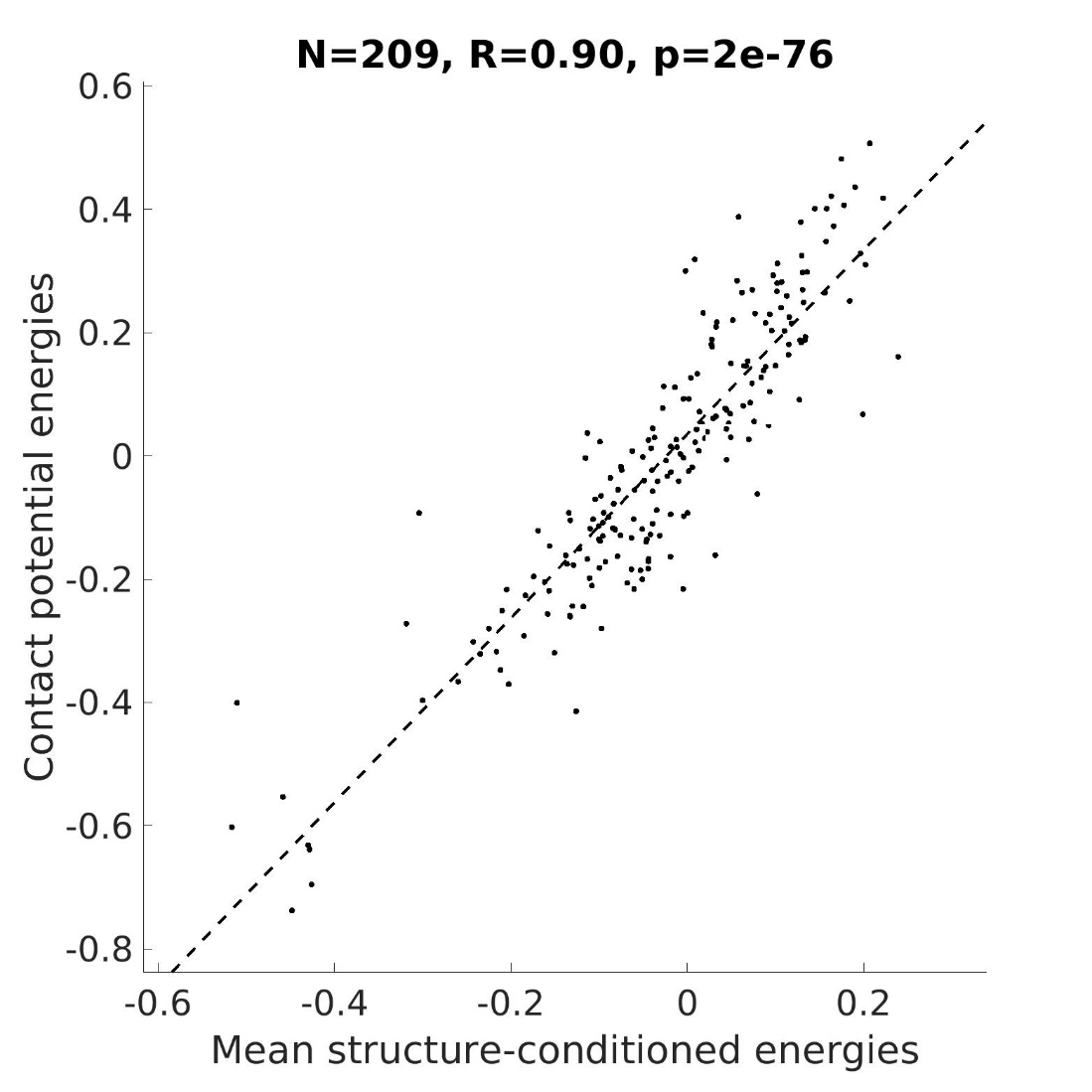


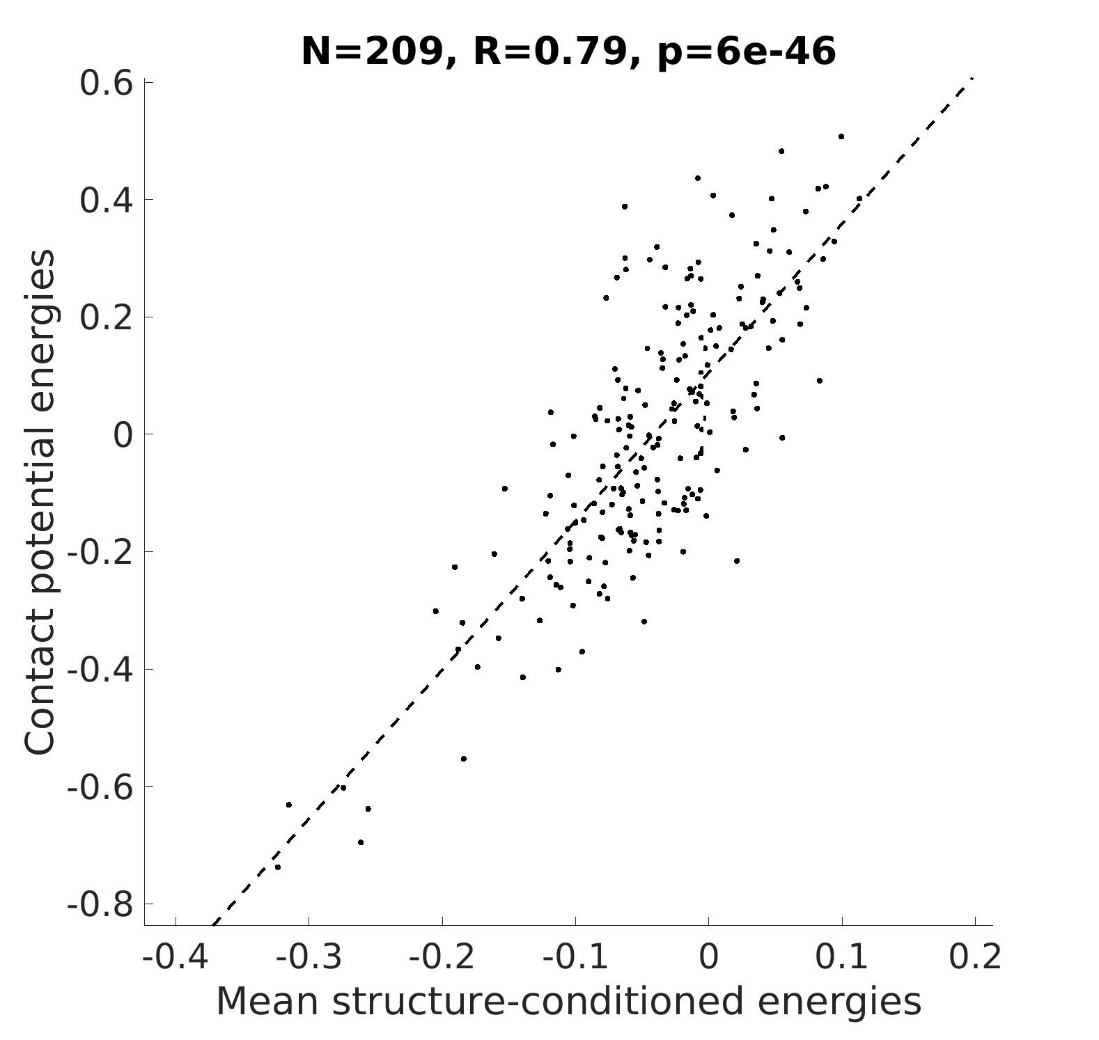


**Fig. S1** Additional information about correlations between SCEs and CEs. **Top**: Distribution of correlation coefficients between each set of SCEs in DB200K and the CEs. The dotted line indicates the mean correlation of R=0.20. **Middle**, **Bottom**: Plot of the averaged, symmetrized SCEs from 1x1- (Middle) or 5x5-motifs (Bottom) vs those from a contact potential. As with Fig. 2A, Cys-Cys is not shown as it occupies points to the far bottom-left (mean SCE of -1.70 (1x1) or -0.88 (5x5)), though its inclusion increases the correlation to R=0.91 (1x1) or R=0.84 (5x5).


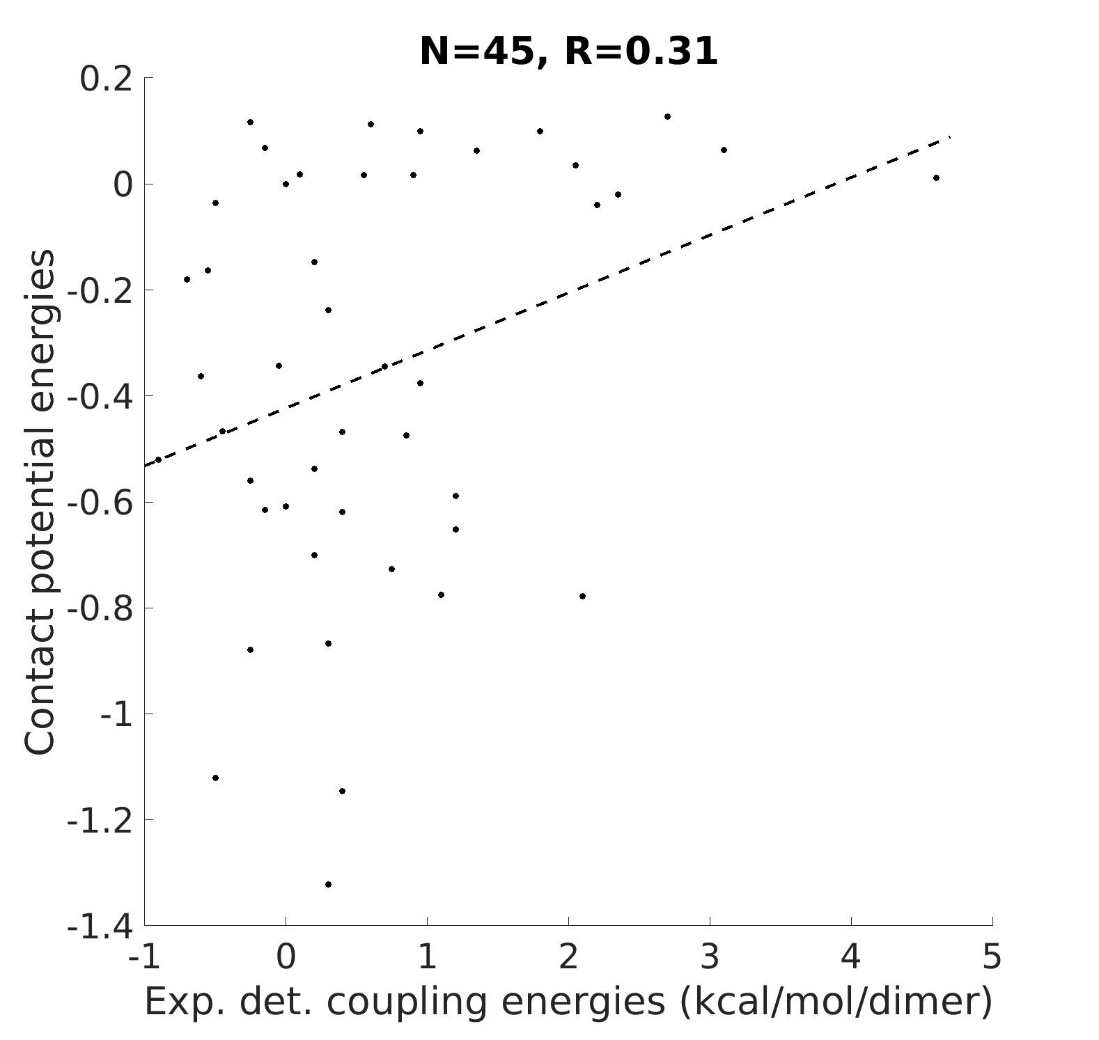


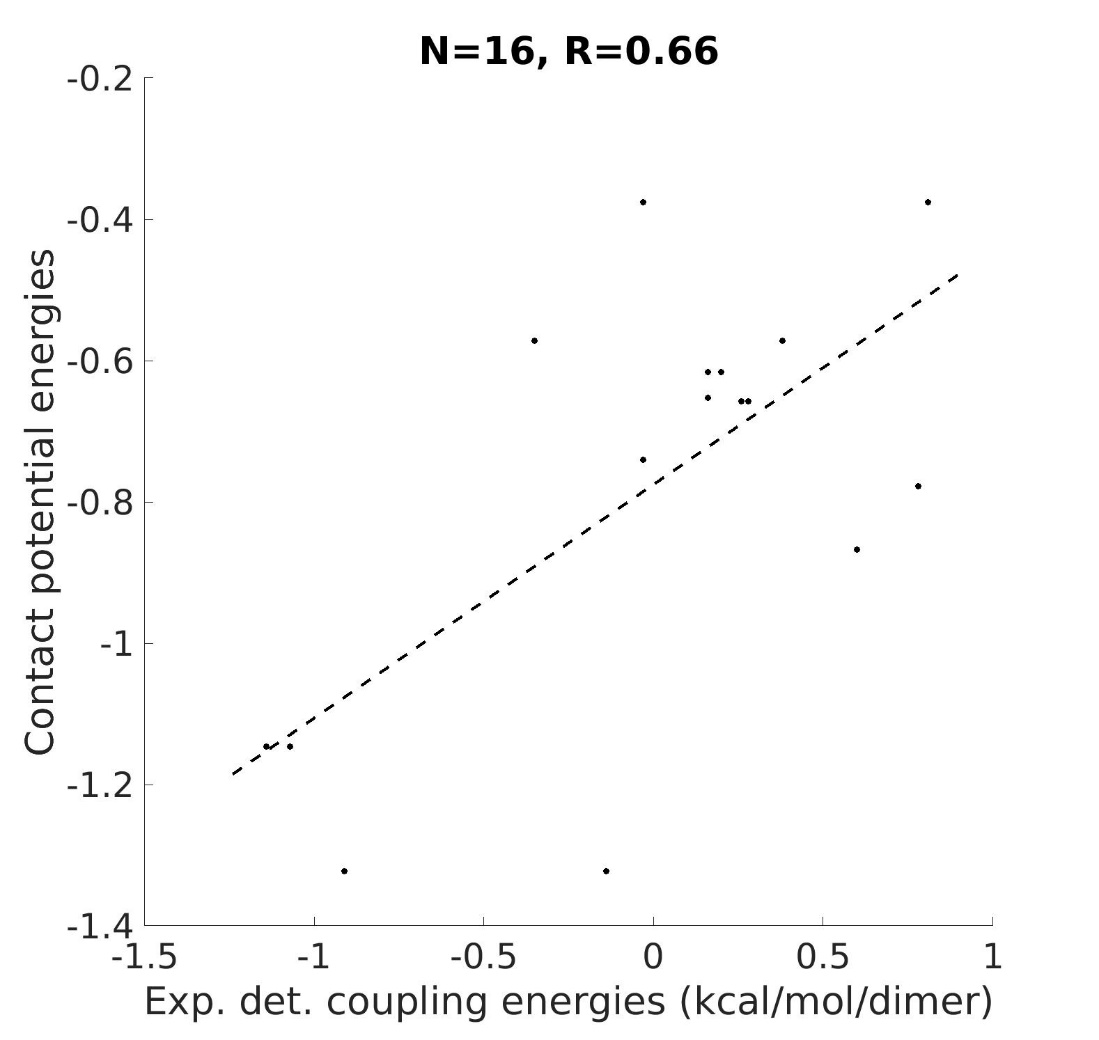


**Fig. S2** CEs vs experimentally determined coupling energies. **Top**, **Bottom**: Correlation between experimentally determined energies vs CEs for a-a' (Top) and g-e' (Bottom) interactions. The dotted line indicates the best linear fit of the data.


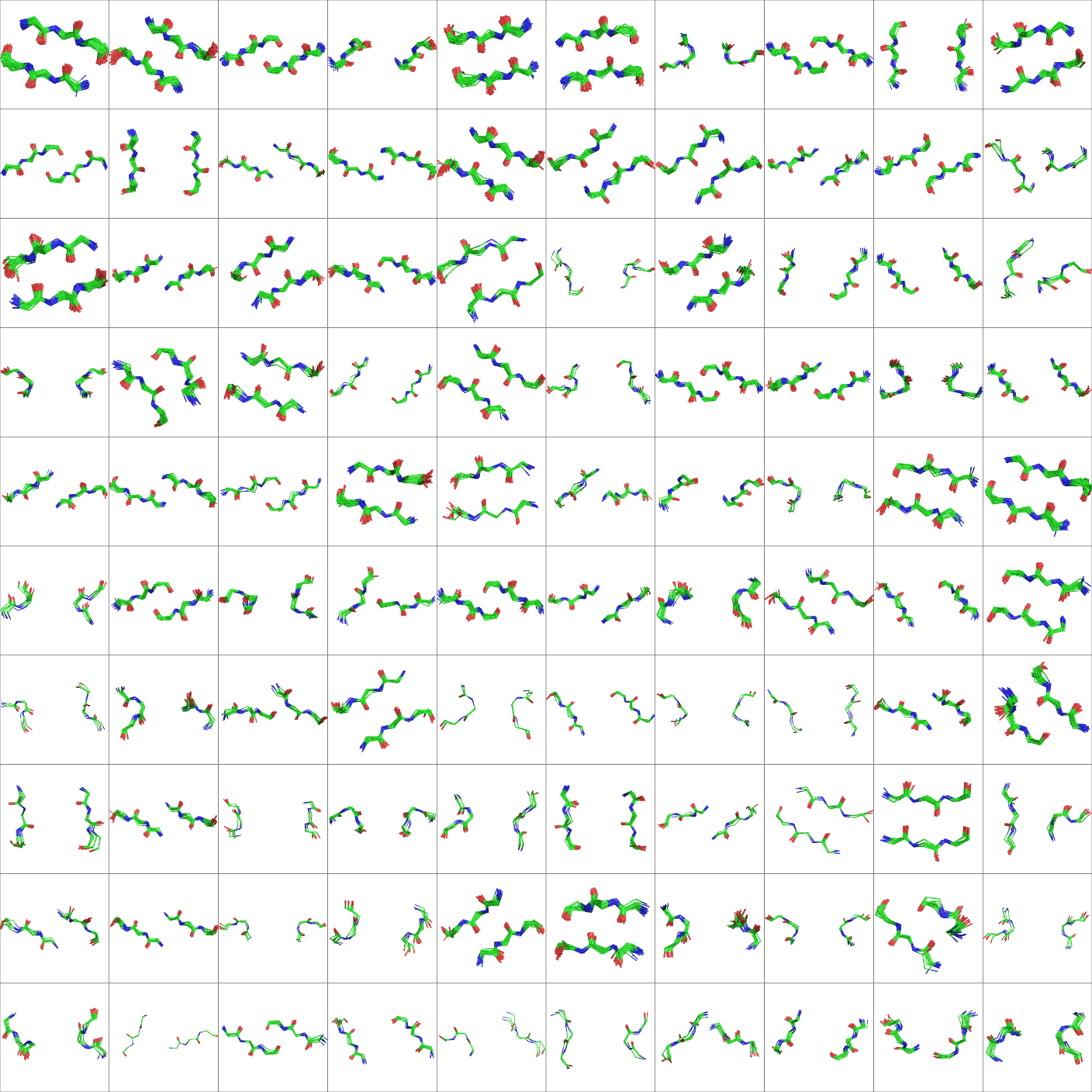


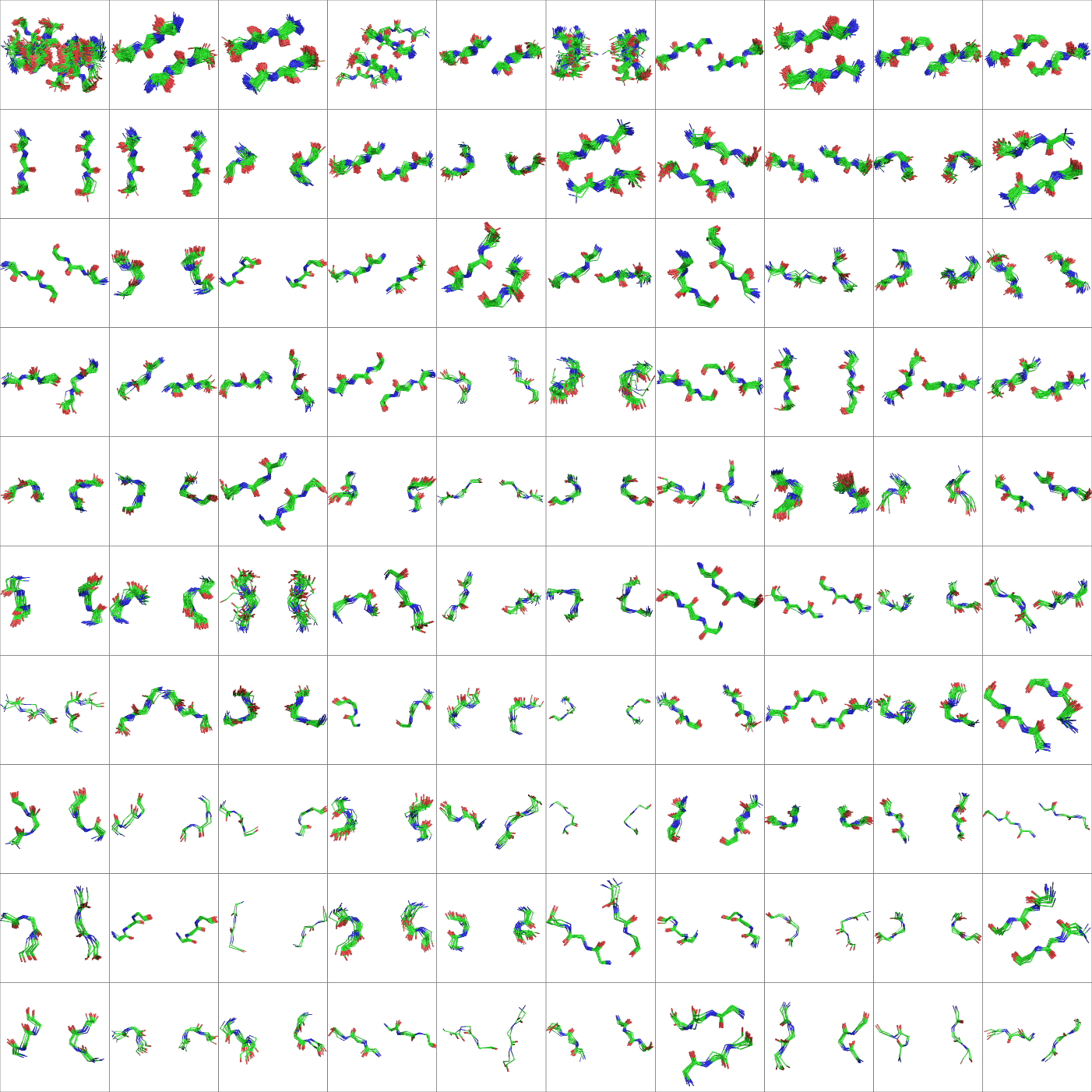


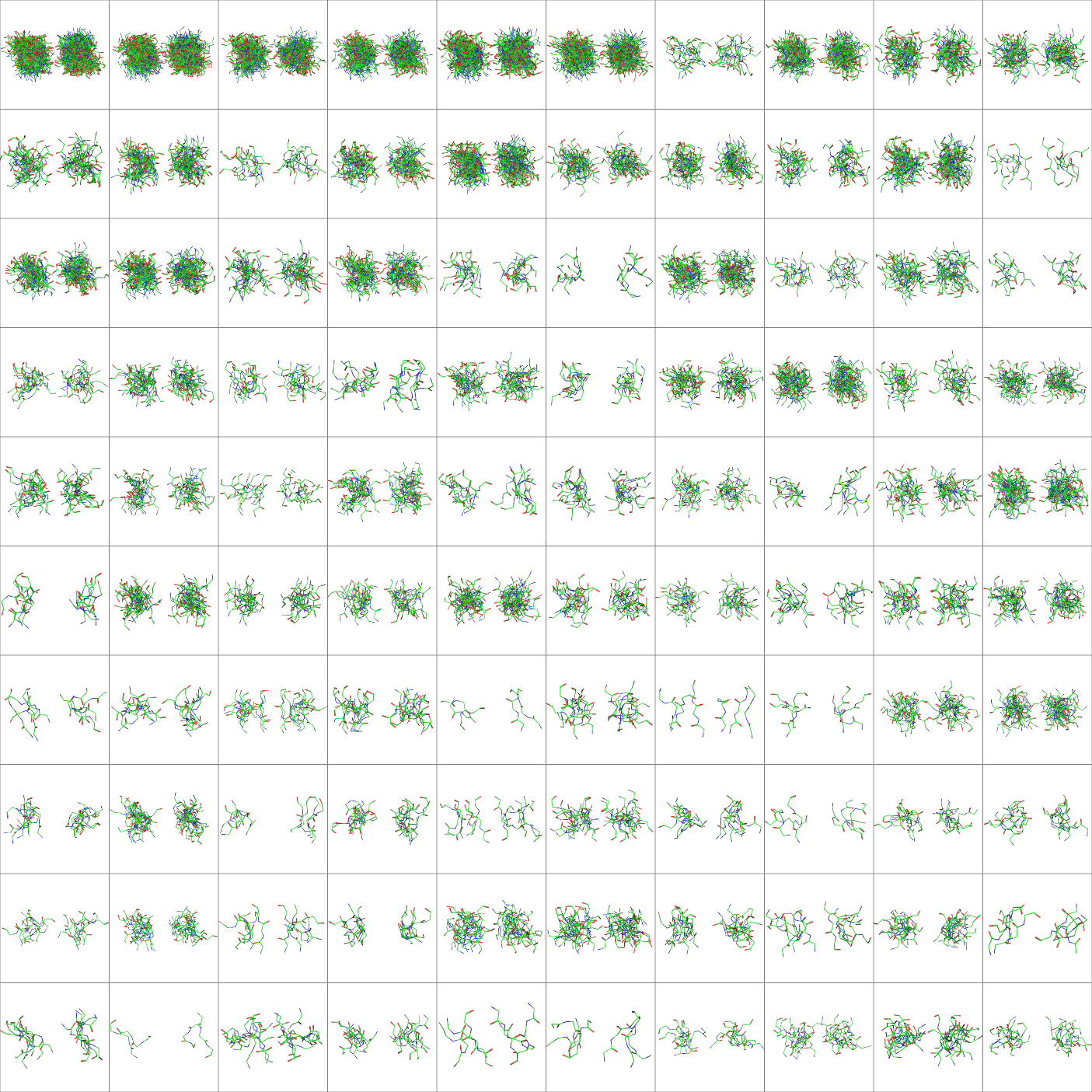


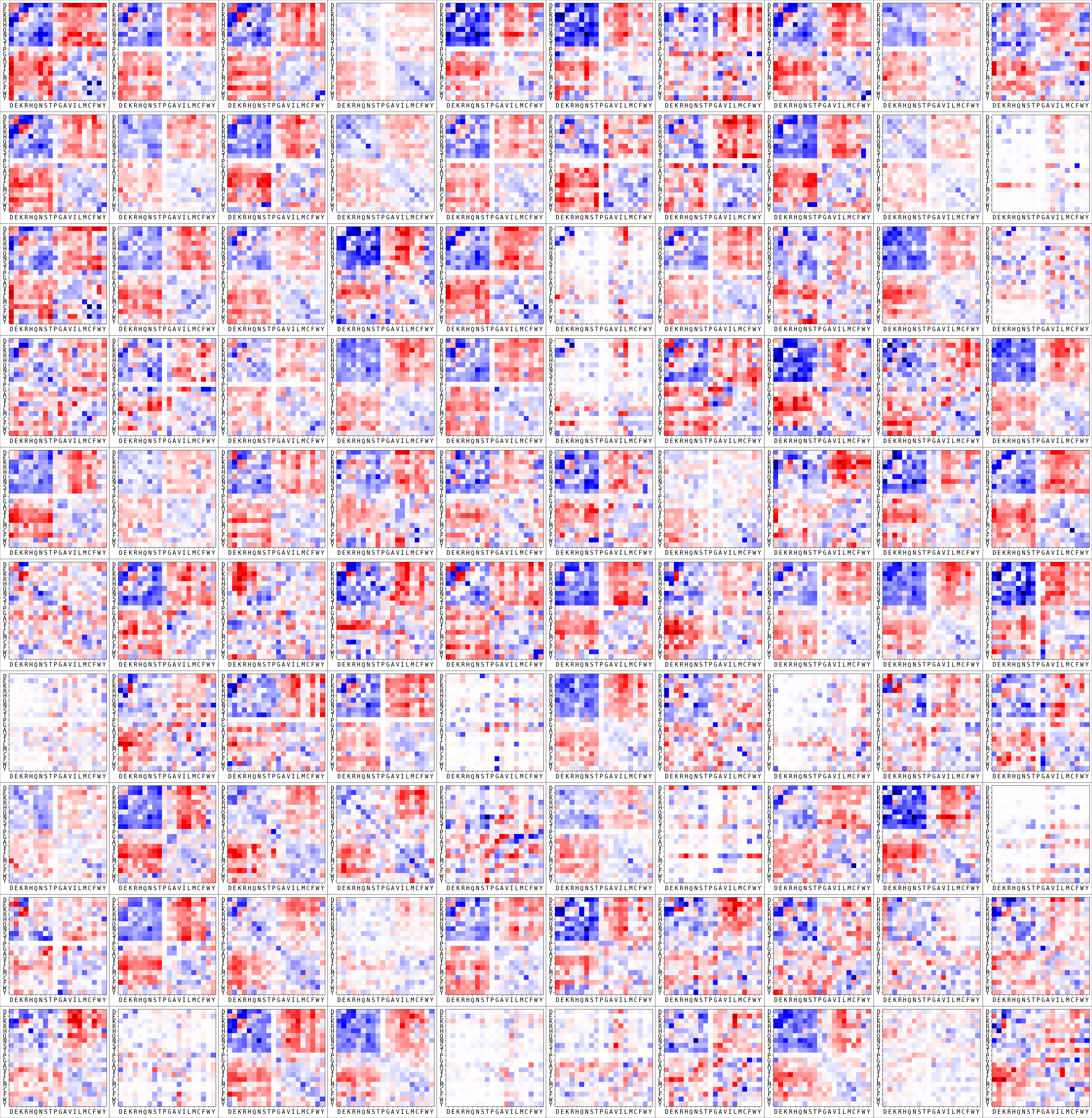


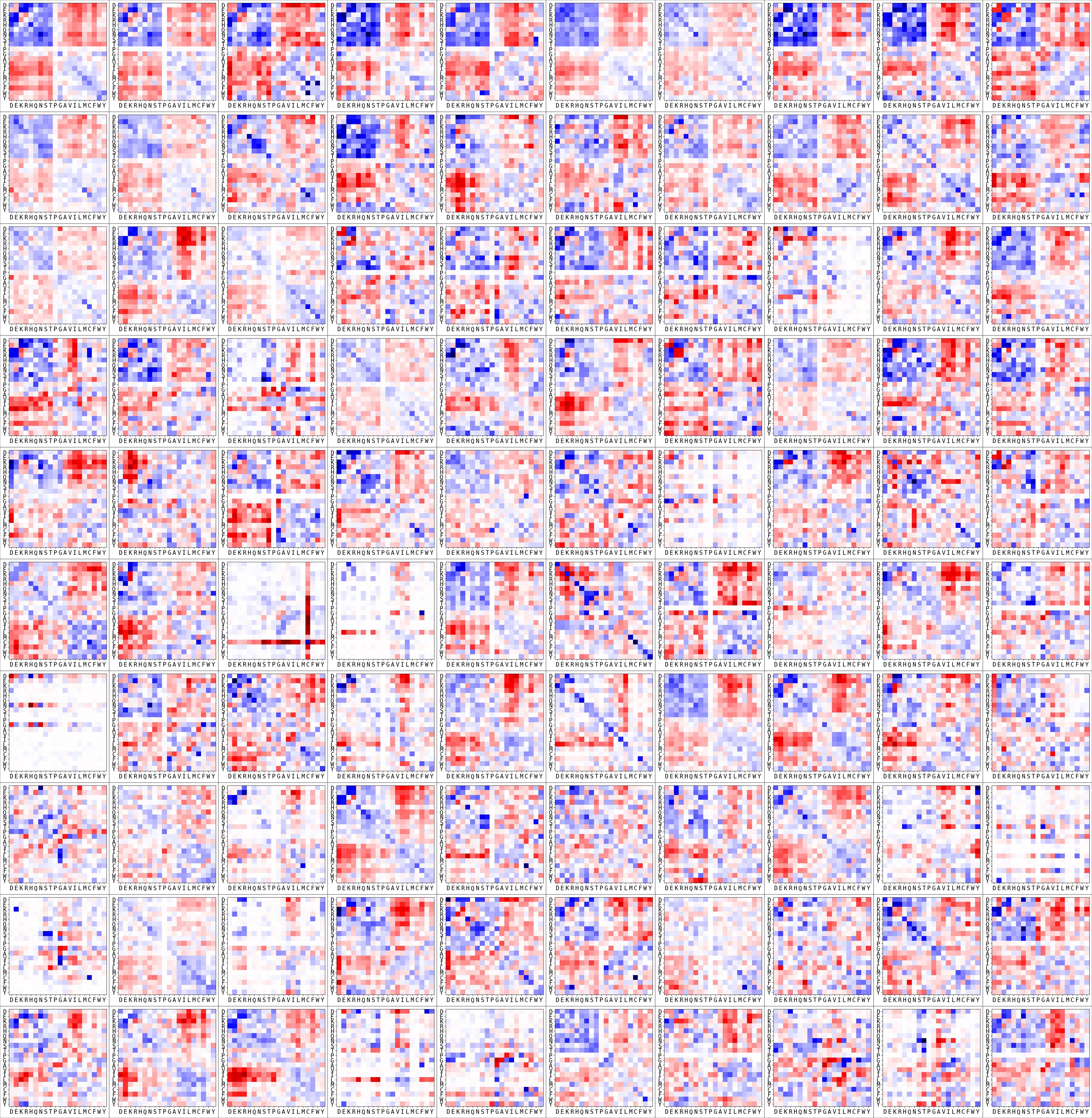


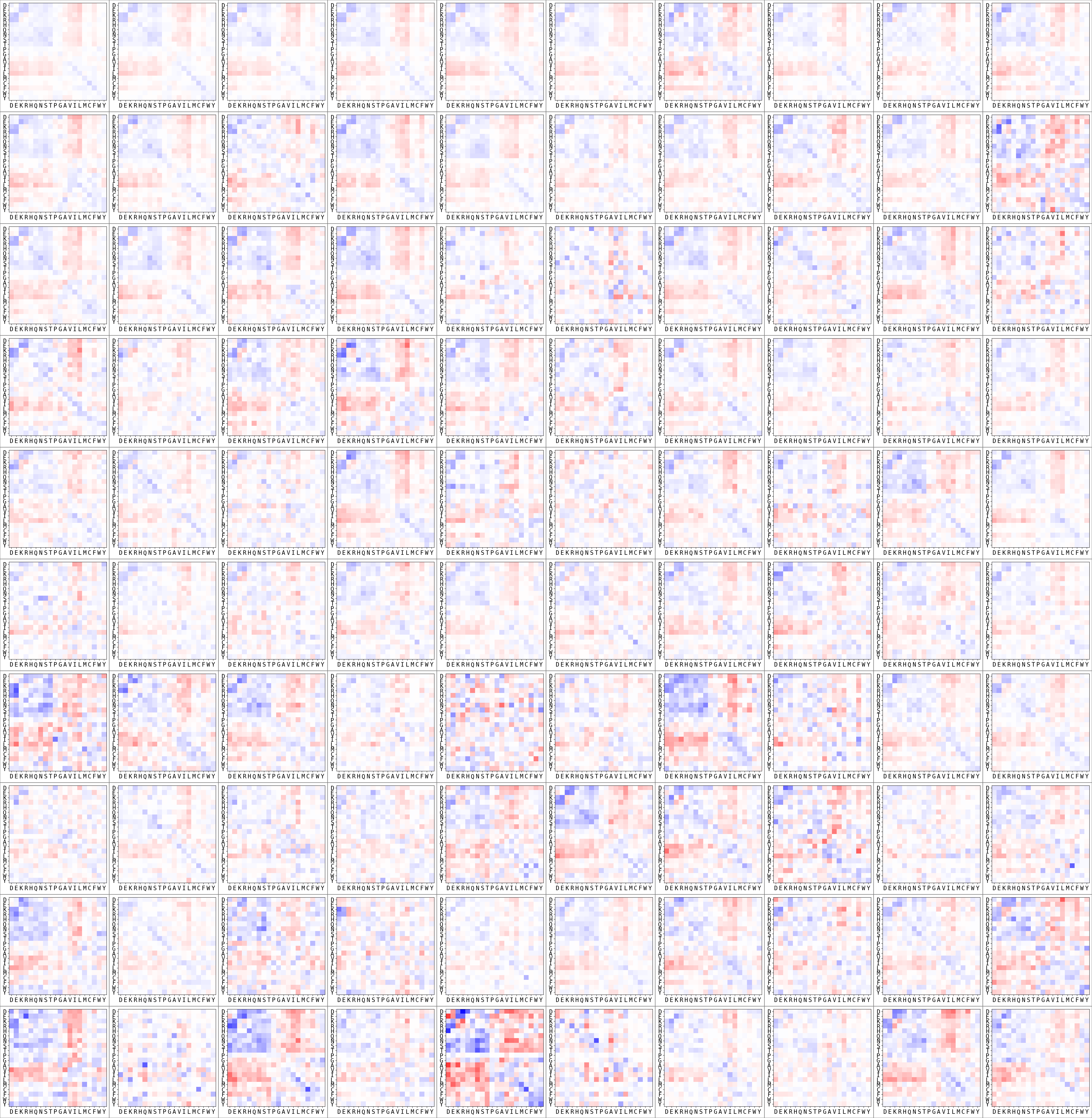


**Fig. S3** Additional clustering visualizations. The first three figures show the fragment ensembles of the top 100 clusters when clustering by structure, energy, or randomly, respectively. The bottom three figures show the mean SCE matrices for these respective clusterings. The color scale is the same as shown in Fig. 5.


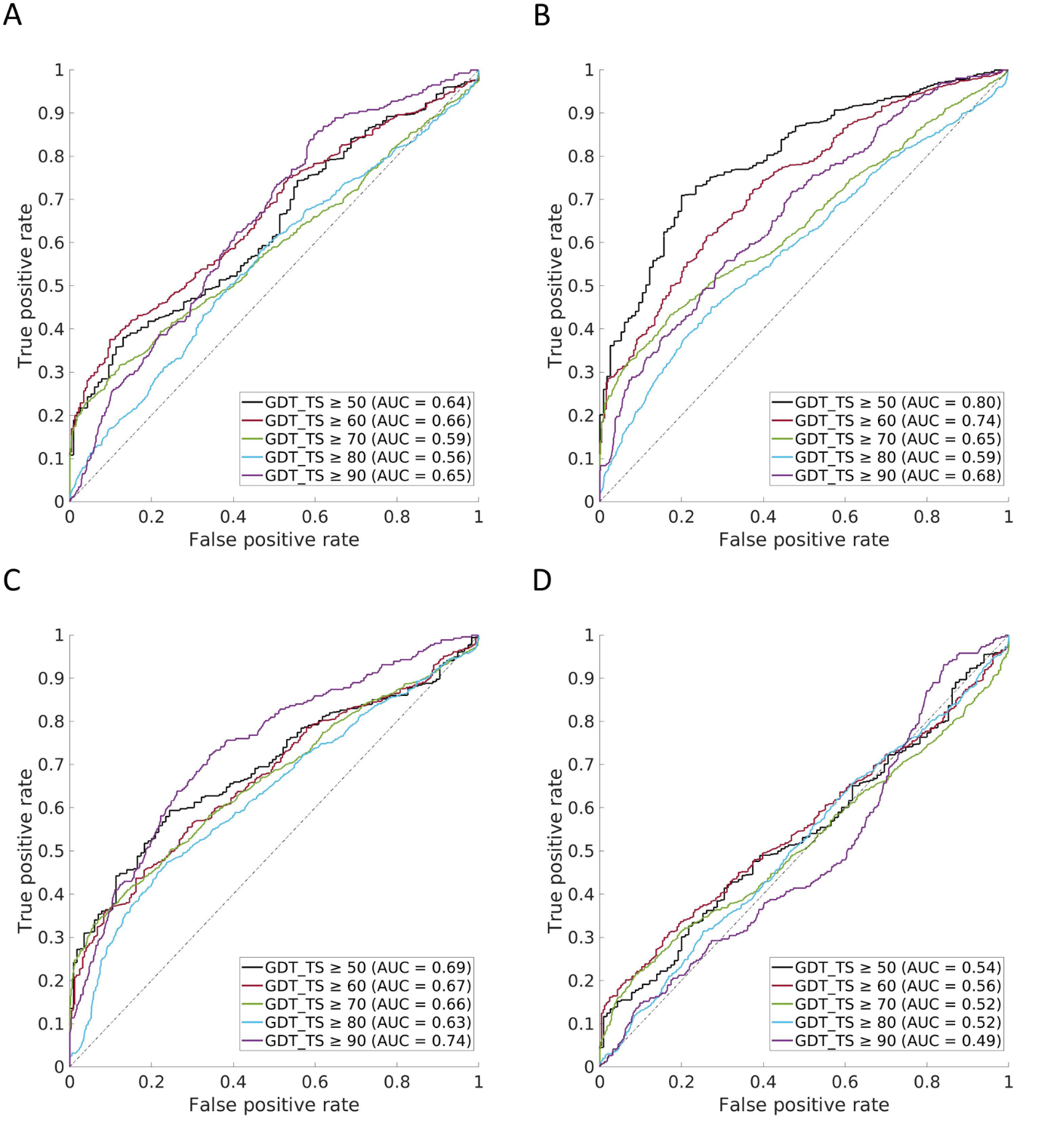


**Fig. S4** Relationship between GDT_TS and statistical energies over a set of predicted CASP models and their corresponding native structures via ROC curves. **A**-**C**: SCEs vs GDT_TS. (A), (B), and (C) correspond to 1x1, 3x3, and 5x5 SCEs. **D**: CEs vs GDT_TS.


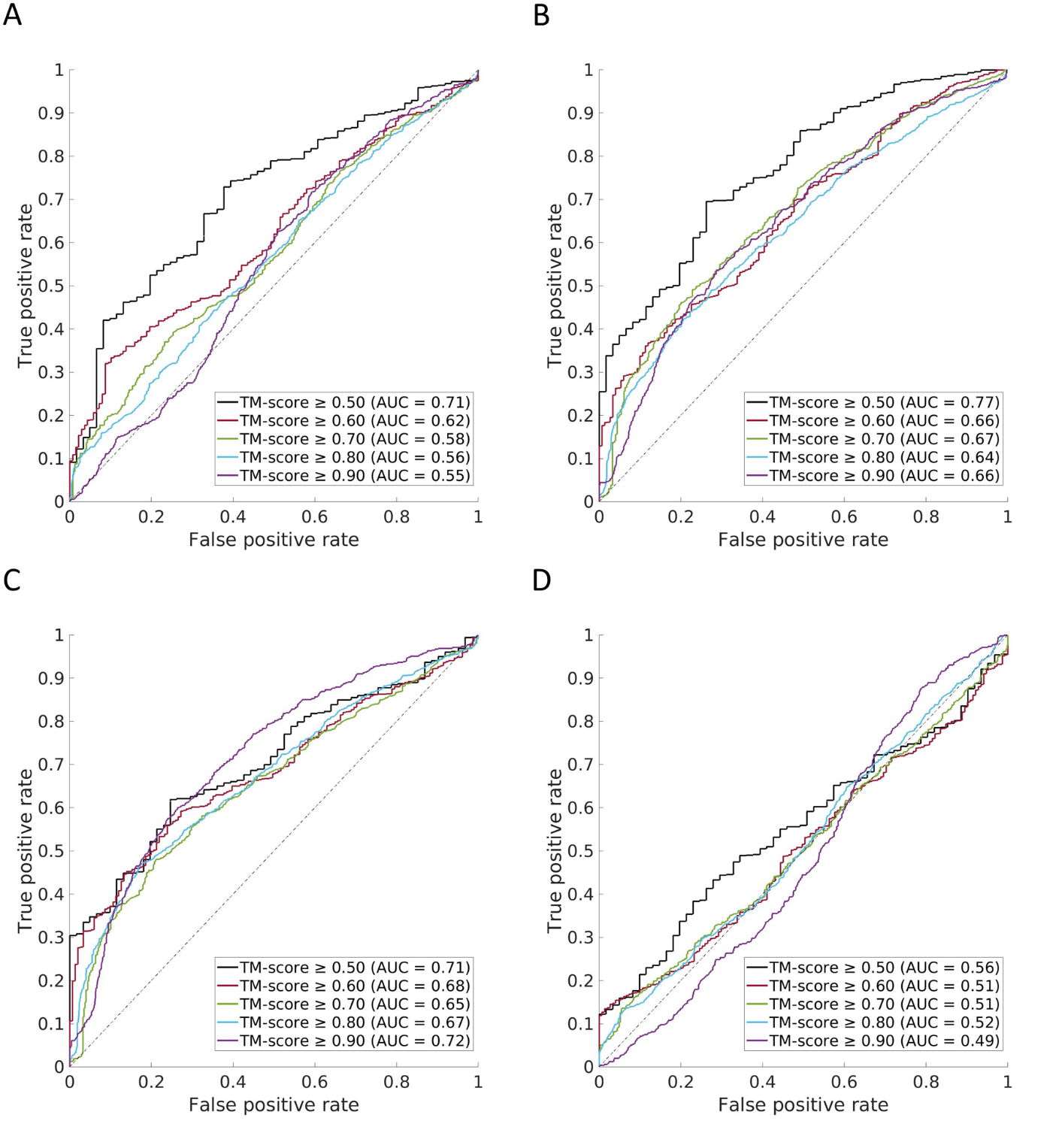


**Fig. S5** Relationship between GDT_TS and statistical energies over a set of predicted CASP models and their corresponding native structures via ROC curves. **A**-**C**: SCEs vs TM-score. (A), (B), and (C) correspond to 1x1, 3x3, and 5x5 SCEs. **D**: CEs vs TM-score.


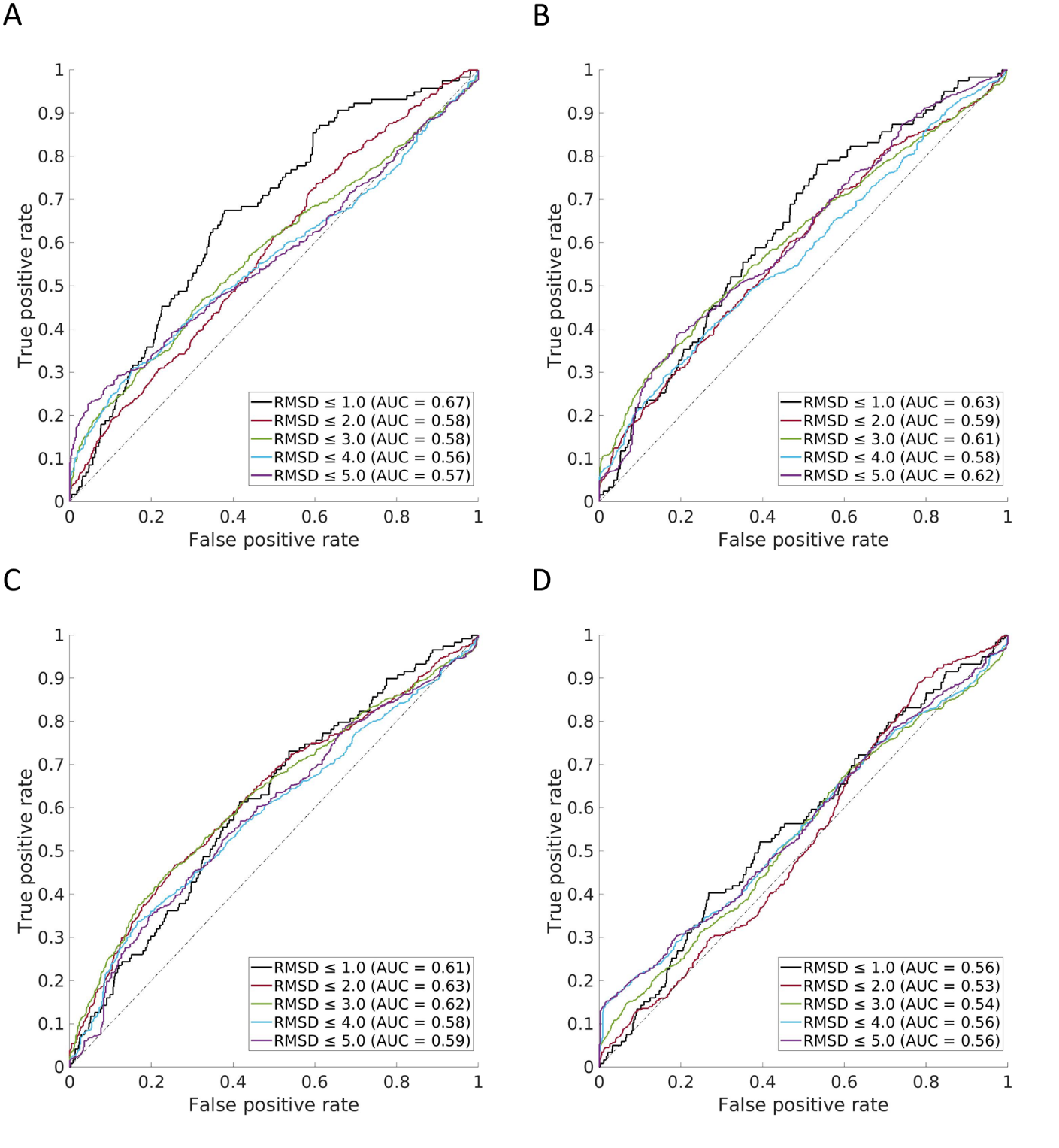


**Fig. S6** Relationship between GDT_TS and statistical energies over a set of predicted CASP models and their corresponding native structures via ROC curves. **A**-**C**: SCEs vs RMSD. (A), (B), and (C) correspond to 1x1, 3x3, and 5x5 SCEs. **D**: CEs vs RMSD.
